# A nanotechnology-based approach to enhance caraway (*Carum carvi* L.) productivity, chemical, biochemical and essential oil under salinity stress

**DOI:** 10.3389/fpls.2026.1814332

**Published:** 2026-06-08

**Authors:** El-Sayed Mohamed El-Mahrouk, Amira Mohamed Tayaa, Mohamed Kadry Gaber, Ekramy Abdel-Moatamed Atef, Giuseppe Cristiano, Danilo Loconsole, Barbara De Lucia, Mohamed A. A. Ahmed

**Affiliations:** 1 Horticulture Department, Faculty of Agriculture, Kafrelsheikh University, Kafr El-Sheikh, Egypt; 2 Plant Production Department, Faculty of Agriculture (Saba Basha), Alexandria University, Alexandria, Egypt; 3 Department of Soil, Plant and Food Sciences, University of Bari “Aldo Moro”, Bari, Italy; 4 Plant Production Department (Horticulture - Medicinal and Aromatic Plants), Faculty of Agriculture (Saba Basha), Alexandria University, Alexandria, Egypt

**Keywords:** *Carum carvi*, enzyme activity, physiological mechanisms, salinity, volatile oil, zinc oxide nanoparticles

## Abstract

Salt stress is an abiotic stressor that adversely affects the growth and productivity of caraway, an important aromatic plant. A randomized complete split-plot design was used to determine the nanoparticles of zinc oxide (ZnO NPs) effects at levels of 0, 0.2, and 0.4 g L^-1^ on fruit yield, chemical and biochemical composition, and essential oil (EO) productivity of caraway plants subjected to saline irrigation water containing 0, 1, 2, 3, and 4 g L^-1^ sodium chloride (NaCl). The results indicated that increasing NaCl concentrations significantly reduced yield traits, relative water content, salinity tolerance index, leaf pigment concentrations, N, P, K, and Zn concentrations, fruits’ total proteins and total carbohydrates and percentage and yield/plant of essential oil (EO) in comparison to the untreated plants. Conversely, proline content, Na% and Cl%, and the activities of catalase, peroxidase, superoxide dismutase, and polyphenol oxidase increased with increasing NaCl concentrations relative to the control. Foliar application of ZnO NPs significantly increased the parameters relative to the untreated control except proline content, Na%, and Cl%, which were in comparison to respective control significantly reduced. Moreover, utilization of ZnO NPs positively affected the traits mentioned above under all NaCl concentrations tested compared to treatments without ZnO NPs. Different combinations of NaCl and ZnO NPs concentrations had varying effects on EO composition. A total of 32 compounds were identified across all treatment combinations, with the highest number (14) observed in the control. The major EO compounds were carvone (up to 49.71%) in the 2 g L^-1^ NaCl + 0.2 g L^-1^ ZnO NPs treatment, limonene (up to 28.68%) in the 2 g L^-1^ NaCl + 0.4 g L^-1^ ZnO NPs treatment, and D-limonene (up to 25.95%) in the 4 g L^-1^ NaCl + 0 g L^-1^ ZnO NPs treatment. These results suggest that the application of ZnO NPs may provide a sustainable approach to caraway cultivation under saline conditions.

## 1 Introduction

Recently, global demand for aromatic and medicinal plants has increased substantially due to their diverse uses in food and medicine. Caraway (*Carum carvi* L.) belongs to the family Apiaceae. It is a significant aromatic species cultivated in Egypt. Its fruits are essentially used in both food and pharmaceuticals. The bioactive compounds of caraway are used for digestive issues, as a tonic, and as an antispasmodic, and they provide carminative properties (Sultan et al., 2014). The main compounds in the caraway dried fruits are 4 - 8% volatile oil, primarily composed of carvone (52%) and limonene (45%) (Olle et al., 2010). The fruits contain organic acids, minerals, sugars, flavonoids, derivatives of coumarins, and approximately lipids (13–21%), fatty oil (22%), nitrogen compounds (25–35%), water (9–13%), proteins (25%) and fibers (13–19%) (Kluszczyñska, 2002). Additionally, Lawless (1992) documented that caraway fruits are a vital source of monoterpenes and are traditionally utilized to treat dyspepsia, intestinal colic, and spasms, and to enhance the flavor of culinary dishes.

Several abiotic stresses, such as high salinity, affect plant survival and growth (de Medeiros et al., 2023). These environmental stresses can impact plants at various stages of growth and trigger complex responses which regulate their morphological and chemical characteristics (Münchinger et al., 2023). Consequently, quality and yield properties may be significantly impacted with pressure (Khare et al., 2020). Salinity causes harmful effects on plants, particularly in arid habitats. Various plant species have different defense mechanisms against abiotic and biotic stressors to protect themselves. These defense mechanisms include modifying ion balance, increasing the antioxidant pool, accumulating compatible solutes, producing nitric oxide, regulating hormonal balance, and regulating gene expression (Van Zelm et al., 2020). They added that there is typically a harmony between ROS production and normal growth conditions.

Soil salinity increases, leading to harmful reactions in plants as reactive oxygen species (ROS) levels rise, thereby inducing oxidative damage to cellular macromolecules. The ROS, such as superoxide anions (O_2_^-^), hydrogen peroxide (H_2_O_2_), and trace amounts of transition metals, also increase the concentration of OH^-^. At the same time, plants conduct detoxification to alleviate the oxidative damage, because the antioxidant enzymes have an essential function that markedly contributes to the plant tolerance in saline stress (Wang et al., 2009) and alleviate the production of ROS via developing defense systems of enzymatic and non-enzymatic antioxidants (Hasanuzzaman et al., 2020). The excessive induction of ROS leads to diminution in photosynthetic efficacy, conductance of stomatal, and rate of net photosynthetic, and impairs the membrane-anchored phospholipids and causes breakdown of membranes and denaturation of lipids (Wani et al., 2020), as well as leading to chlorosis and senescence of premature leaves in plants Therefore, metabolism and physiology of plants conversely are affected by salt stress, thus retarding the growth stages and production of plants (Kumar et al., 2022). Also, salt stress significantly retarded vital fluorescence-based photosynthetic metrics, changing essential processes such as transport of electrons, storage of energy and the mechanism of photoprotection (Faizan et al., 2021).

Plants tolerate salinity stress by increasing osmolyte concentrations, such as sugars, polyamines, and proline (Sharma et al., 2012). Additionally, antioxidant molecules, such as glutathione, and proteins can help control ROS levels, thereby improving resistance to salinity stress. Additionally, soil salinity increases Na+ and Cl^-^ levels in plants, thereby raising the Na+/K+ ratio, which ultimately affects the activities of regular ions in plants (Singh et al., 2014). Many plants have evolved various strategies to avoid these threats. Among these, the first strategy is the maintenance of homeostasis via osmotic regulation, which transports excess Na+ ions to the vacuole, and the second is the synthesis of osmolytes to cope with this condition (Rahneshan et al., 2018). Furthermore, a high K+/Na+ ratio plays an important role in maintaining membrane integrity and osmotic and turgor pressures. It also benefits enzyme activation and tropism (Rahneshan et al., 2018).

Nanotechnology has been applied in several ways, including improving crop productivity, reducing the use of fertilizers and pesticides, and indirectly protecting the environment (Rui et al., 2016). Nanotechnology applications offer significant benefits to the horticultural and food industries (Swarnapriya and Vaibhao Ramesh, 2020). Nanoparticles (NPs) of nutrients that ranged from 1–100 nm in size, have several benefits, including enhanced plant growth, nutrition and plant productivity (Cele, 2020). From the NPs, nanoparticles of zinc oxide (ZnO NPs) are considered sustainable nanoparticles owing to their essential physico-chemical and biological characteristics. These include biocompatibility, environmental safety, ease of preparation, and non-hazardous nature, which have led to their overuse across various scientific applications (Alhujaily et al., 2022). Moreover, owing to their high reactivity, ZnO NPs supply plants with an adequate Zn form, thereby alleviating zinc deficiency, particularly given the low solubility of soil zinc resources (Seleiman et al., 2023). The same authors added that the influence of ZnO NPs on plants may vary with the species tested, their growth stage, and nanoparticle characteristics. Factors such as pH, physicochemical soil characteristics, and plant species resistance percentages can affect soil Zn availability and its effects on plant species diversity (García-Gómez et al., 2018). The efficiency of NPs of minerals in nutrient uptake, distribution and accumulation within plants is significantly impacted by intrinsic factors like particle size and surface coatings, also, extrinsic factors such as soil texture, pH, and organic matter content as well as application method plays a critical function (Ma et al., 2018). Zinc interacts with several soil minerals, especially N, P, Na and Cl (Loneragan and Webb, 1993; Ismail et al., 2014). Minerals level, pH, EC, organic matter and water available of soil are often linked to soil texture. It means that factors that cause nutrient balance or imbalance in the soil can lead to increase in ZnO NPs foliar application efficacy (in case of nutrient imbalance and soil zinc deficiency and non-suitable level for plant) or decrease in ZnO NPs foliar application efficacy (in case of nutrient balance and soil zinc available and suitable level for plant). Moreover, environmental conditions such as high or low temperature and relative humidity, wind speed, photoperiod which affect respiration, transpiration rate, stomatal opening, closure, conductance and photosynthesis rate can also positively or negatively affect ZnO NPs foliar application efficacy. Therefore, foliar spraying with ZnO NPs is the most effective method for addressing micronutrient deficiencies or their decline in plant species (Adrees et al., 2021). Through spraying, plants readily and immediately take up NPs, regardless of the application method, from soil elemental fertilizers (Kah et al., 2018).

Zinc is one of the most essential micronutrients, affecting growth, development, and productivity, and it mediates plant responses under stress conditions (Broadley et al., 2007). Zn is a cofactor for several key antioxidant enzymes and plays indirect roles in RNA and DNA synthesis (Song et al., 2015). Gauba et al. (2023) demonstrated that ZnO NPs increased enzyme activity, enhanced photosynthetic performance, increased seed yield, and increased the levels of antioxidant and osmoregulatory compounds. However, the mechanisms of defense related to ZnO NPs against salt stress include modifications in the structure of the root system, compartmentalization, phytohormone modulation and synthesis of osmolytes, adjustment of Na+ homeostasis, antioxidant activity stimulation, upregulation of stress tolerance genes, and cohabitation with halotolerant rhizobacteria (Ramasamy and Mahawar, 2023). Many areas in Egypt are affected by salinity and irrigation water shortages, particularly those newly irrigated with drainage water, which contains high salt levels in most locations. Therefore, the current investigation aimed to improve fruit yield, chemical and biochemical composition, and essential oil productivity of caraway (*Carum carvi* L.) by foliar spraying with ZnO NPs under saline irrigation.

## 2 Materials and methods

A pot experiment was conducted at the Farm of Faculty of Agriculture, Saba Basha, Alexandria University, Alexandria, Egypt, through the 2023/2024 and 2024/2025 seasons to investigate the response of caraway (*Carium carvi* L.) plants to different levels of foliar spraying with ZnO NPs under salinity stress. The average meteorological data were recorded during the experimental period (October to April) at the Alexandria location (Altitude: 32 m; Latitude: 31.36°N; Longitude: 29.95°E), Egypt. The average of Max. temperature (22.6 and 22.7 °C), Min. temperature (13.4 and 15.1 °C), relative humidity (62.2 and 60.6%), wind (15.1 and 14.5 Km/day), rainfall (4.05 and 3.83 mm) and solar radiation (15.97 and 16.31 MJ/m^2^/day) in 2023/2024 and 2024/2025 seasons, respectively.

### 2.1 Soil properties

The soil sample was crushed after air-drying using a mortar and pestle. A stainless-steel test sieve was used to divide the soil sample into fractions of less than 2 mm (Cools and De Vos, 2011). The particle-size distribution of the soil material was assessed using the hydrometer method (US 21 CFR 1040.10 and 1040.11, USA) as described by Gee and Bauder (1986). A 1:5 mixture of dried soil (20 g) and distilled water (100 mL) was assessed to determine the soil’s chemical characteristics, and the mixture was incubated for 24 h. After that, the extract was filtered. The following determinations were made in the filtered extract: the EC of the soil was measured using an EC meter (MI 170, SZ, Egged, Hungary, Italy) (Jackson, 1973). Additionally, the procedures of Jackson (1973) were applied to measure magnesium, calcium, and chloride levels (ENREF-40). The procedure of Nelson and Sommers (1996) was used to quantify total carbonate and organic matter (OM). The micro-Kjeldahl (DNB, 1500 NPS.N.33848, made in Spain, RAYPA) procedure was used to estimate the level of accessible nitrogen (N) (Bremner and Mulvaney, 1983). Available phosphorus was determined using the Olsen and Sommers (1982) method. An atomic absorption spectrophotometer (AAS) was used to assess zinc (Zn ^#^) availability (Page et al., 1982). Sodium and potassium were determined with the method of Black et al. (1965) by a PSC 7 flame photometer (JENWAY, Staffordshire, UK). The soil pH was measured in suspension (1:2.5, soil:deionized water) after 30 minutes using a pH meter (JENWAY 3510, Staffordshire, UK) (Black et al., 1965). Whereas the soil EC at the end of the experiment is 1.70, 3.28, 4.83, 6.91 and 7.95 ds/m after application of NaCl at 0,1,2,3 and 4 gL^-1^, respectively. Physicochemical properties of the used soil are displayed in (**Table 1**).

**Table 1:** Physicochemical properties of the soil used.

Physical parameters		Chemical parameters					Available macronutrients (mgkg^-1^)				
Clay Sand Silt	65.82% 24.18% 10.00%	pH (1:2.5)		Organic matter %		EC (ds/m)	N		P	K
		7.70		2.30		1.72	788		27.3	50.4
Soil texture: clay		Cations (meq^-1^)					Anions (meq^-1^)			Zn (ppm)
		Na^+^	K^+^	Mg^++^	Ca^++^	CO^--3^	Cl^-^	HCO^-3^	SO^—4^	
		9.2	0.80	4.40	2.20	0.0	4.60	2.90	1.42	435.20

### 2.2 Sowing of seeds

Caraway seeds were obtained from the Medicinal and Aromatic Plants Department, Horticulture Research Institute, Agriculture Research Centre, Agriculture Ministry, Egypt. The seeds were sterilized in a 5% commercial Na hypochlorite solution for 5 min, then sown on 1 ^st^ October in 2023 and 2024 in pots with a diameter of 40 cm and a height of 32 cm, with 4 seeds per pot, using 9 kg of soil. Germination was nearly complete after 14 days of sowing. The plants were thinned after 30 days from sowing (2 plants/pot), and after ten days, the plants were thinned to one plant/pot. Regular irrigation was done by tap water until the start of the study.

### 2.3 Layout of experiment

The experimental layout was a randomized complete split plot design (Gomez and Gomez, 1984) in 3 repetitions, where the main plots were water salinity treatments (NaCl levels), while the sub-plots were ZnO NPs treatments. Each repetition comprised 15 treatments (5 NaCl levels × 3 ZnO NP concentrations). Each treatment comprises 3 pots per repetition; thus, each treatment includes 9 pots across 3 repetitions. This indicates that the experiment included 135 plants per season.

### 2.4 Irrigation application

Sodium chloride (99.5% purity) was obtained from Elgomhorya Company in Alexandria and used as a salinity source. After the second thinning (40 days from seed sowing), the plants were irrigated, with saline water contained NaCl at different used concentrations every 3 days three times and one time with tap water (pH 7.2; EC 0.59 dSm^-1^), to prevent salt accumulation. The irrigation water was increased from 0.75 to 1.25 L/pot of in irrigation with increasing the plant age thought the experimental period. The plants were placed under a white plastic umbrella to protect them from rainfall.

### 2.5 Nanoparticles of zinc oxide

ZnO NPs at 0.0, 0.2 and 0.4 gL^-1^ were used three times as foliar application after 40, 60 and 80 days from the seed sowing.

#### 2.5.1 Zinc oxide nanoparticles synthesis

**Figure 1:**
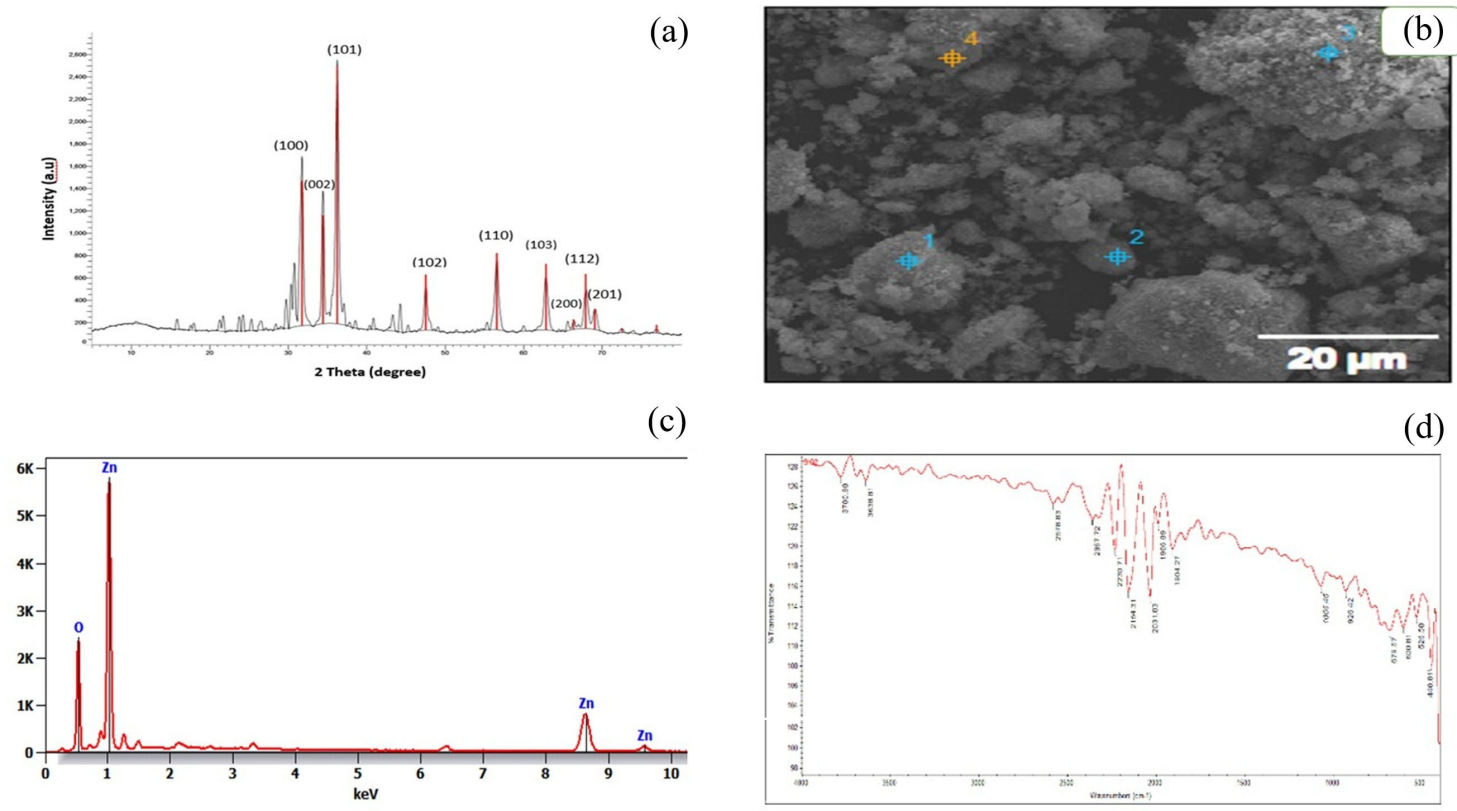
**(a)** ZnO NPs XRD; **(b)** ZnO NPs SEM; **(c)** ZnO NPs ADX; and **(d)** ZnO NPs FTIR.

Zinc oxide NPs were obtained using Ali et al. (2017) procedure. Where the combination of 50 mL of 2m NaOH and 100 mL of 1 mM Zn (CH_3_COO) _2_, 2H_2_O were carefully dropped and stirred continuously for 2 h. At room temperature (25 ± 2 °C), centrifugation (model 58 10r, Eppendorf Corporation, Hamburg, Germany) was performed at 9508 rpm for 5 min to collect the white precipitate. To displace the impurities, it was washed 3 times with deionized water. Throughout the night, ZnO NPs were dried at 60 °C in an incubator (Thomas Scientific, Swedesboro, NJ, USA). The ZnO NPs powder pattern was recorded. The step-scan spanned the angular range from 20 to 80 degrees with a step size of 0.02 degrees. The crystallite size was determined using the Scherrer equation (D = Kλ/B cos θ). Where the size of crystallite (D), the X-ray radiation wavelength (h), (K) the constant (0.94), the line width at half the peak’s greatest intensity (B), and the diffraction angle (QB). **Figures 1a-d** shows SEM, FTIR, XRD, and EDX of ZnO NPs. Fourier transform infrared (FTIR) absorption spectroscopy (THERMO NICLOT, 50 UK) was employed to assess the nanoparticles’ chemical bonding. The shape of the powder and elemental content were characterized by scanning electron microscopy (SEM; JEOL JSM-6360 LA, Tokyo, Japan) during the elimination process (Naddaf et al., 2020). An X-ray diffraction XRD device (Panalytical Emperian, Istanbul, Turkey) was one approach for explaining crystalline materials. It provides specifications for phases, preferred crystal orientations (texture), and other structural characteristics, such as average grain size, strain, crystallinity, and crystal defects (Pandey et al., 2021). Energy-dispersive X-ray spectroscopy (EDX; JEOL JSM-5300, USA) is a technique for elemental analysis in electron microscopy that uses the characteristic X-ray emission to identify the elements in the sample (Scimeca et al., 2018). Zinc oxide NPs specification and acute toxicity are shown in **Table 2**.

**Table 2:** Zinc oxide NPs specification and acute toxicity.

	Zinc oxide nanoparticles
	Specification
Appearance	white powder
Average particle size	20nm
Morphology (Shape)	Spherical.
Surface area	2.7534 m^2^ g^-1^
Average pore radius	40.5965 nm
Total pore volume	0.042062 cm^3^ g^-1^
Chemical Composition	Zn = 80.34% O = 19.6
Acute toxicity	
The lethal dose 50 (LD50) of intravenously administered	0.3 mgkg^-1^ in mice
The LD50 of intratracheal instillation	493.85 μgkg^-1^

### 2.6 The used treatments

Main plots (NaCl levels): 0 (control: irrigation with tap water) and NaCl at 1,2,3and 4 gL^-1^.

Sub-plots (ZnO NPs concentrations): 0 (control: distilled water), 0.2 gL^-1^ ZnO NPs, and 0.4 gL^-1^ ZnO NPs.

### 2.7 Studied characteristics

At the beginning of flowering stage on 28/1/2024 and 5/2/2025, the following parameters were measured.

#### 2.7.1 Determination of leaf relative water content

Fresh samples of leaves (0.5 g/sample) were immersed in deionized water at a ratio of 1:10 (w/v) for 12 h at 25 °C. After soaking, excess surface water was gently removed using filter paper, and the saturated) Turgid(weight was registered. Subsequently, a leaf sample was oven-dried at 105 °C to a constant weight to remove free water, followed by further drying at 130 °C for 2 hours to remove bound water (Barrs and Weatherley, 1962).


$$\text{RWC}\left(\%\right)=\frac{\text{Fresh weigh}-\text{dry weight}}{\text{Turgid weight}-\text{dry weight}}\times 100$$

#### 2.7.2 Salinity tolerance index

After measuring the longest root length, the STI was performed using the method of Hanif et al. (2017).


$$\text{STI}=\frac{\text{The longest root length of the treated plant}}{\text{The longest root length of the control plant}}\times 100$$

#### 2.7.3 Leaf pigments

The content of leaf chlorophyll a, chlorophyll b, and carotenoids was determined according to Arnon (1949) procedure. A fresh leaf sample (1g) was homogenized in 80% acetone, then centrifuged at 5000 rpm for 5 min. The supernatant was brought to 100 mL in the volumetric flask. The absorbance (O.D.) of the extracted solution was measured at 480, 510, 645, and 663 nm. From these records, levels of chlorophylls and carotenoids were measured with the following formula/equation:


$$\text{Chlorophyll}\left(\text{a}\right)\text{mg/g FW}=\frac{12.7\left(\text{AR}663\text{R}\right)-2.69\left(\text{AR}645\text{R}\right)x\text{V}}{1000}\times \text{W}$$

$$\text{Chlorophyll}\left(\text{b}\right)\text{mg/g FW}=\frac{22.9\left(\text{AR}645\text{R}\right)-4.68\left(\text{AR}663\text{R}\right)x\text{V}}{1000}\times \text{W}$$

$$\text{Carotenoids mg/g FW}\frac{7.6\left(\text{AR}480\text{R}\right)-1.49\left(\text{AR}510\text{R}\right)x\text{V}}{1000}\times \text{W}$$

Where, A= Absorbance at specific wavelengths.

V = Final volume of chlorophyll extract in 80% acetone.

W = fresh weight of tissue extracted.

#### 2.7.4 Leaf chemical composition

The leaves were dried at 75 °C for 72 h in a drying chamber until a constant weight was reached (Tendon, 1993). To obtain a homogeneous powder, the leaf materials were ground in a metal-free mill (Ika Werke, M 20, Germany). The mixture of 0.2 g sample and sulfuric acid (95%, 5 ml) was heated by a sand hotplate for ten minutes. Subsequently, perchloric acid (0.5 mL) was cautiously added, and the heating continued until a clear solution was obtained. After the solution and filtrate cooled, they were diluted to 50 mL (Evenhuis and de Waard, 1980). The percentages of nitrogen, phosphorus, and potassium were achieved using a modified micro- Kjeldahl method (Chemists and Horwitz, 1990), spectrophotometer (GT 80+, UK) (Murphy and Riley, 1962) and an atomic absorption spectrophotometer (Avanta E, GBC, Victoria, Australia) (Cottenie et al., 1982), respectively. The procedures of Jackson (1973) were used to estimate the percentage of Zn. Additionally, the Jackson (1973) technique was employed to determine the percentages of chloride (Cl) and sodium (Na) using a flame photometer.

#### 2.7.5 Leaf proline content

Leaves fresh weight samples (0.5g/sample) were homogenized in 10 mL of 3% aqueous sulfosalicylic acid then the samples were agitated for one hour ahead of filtration through filter papers (2 Whatman., No.1), then 2 mL of the extract was reacted by 2 mL of ninhydrin acid and 2 mL of glacial acetic acid followed by 1 h. of heating at 100 °C in a water bath. Subsequently, the samples were rapidly cooled in an ice bath. 4 mL of toluene was used to extract the reaction mixture, which was then heated to room temperature and aggressively stirred for 20–30 seconds. The absorbance was evaluated at 520 nm by a spectrophotometer (Model SM 1200 UV-Vis, AZZOTA Corporation, Claymont, DE, USA). Proline concentration was estimated from a standard curve, as described by Bates et al. (1973).


$$\mu \text{M proline/g FW}=\frac{\text{mg proline}\times \text{mL toluene}}{115.5}÷\frac{\text{g sample}}{5}$$

#### 2.7.6 Enzymatic activities

The caraway leaf extract was prepared for enzyme activity determination: 1 g of fresh-frozen leaves was ground in liquid nitrogen. Subsequently, a cool extraction buffer (3 mL of 50 mM potassium phosphate; prepared by mixing monopotassium phosphate and dipotassium phosphate at pH 7.5) was used to extract the material. The supernatant obtained by centrifuging the extract at 12,000 rpm and 4 °C for 30 minutes was used to prepare the crude extract. The supernatant was stored at -80 °C for subsequent measurement of antioxidant enzyme activities (Shams et al., 2016). All absorbance values were determined by a UV^-1^900 BMS (Waltham, MA, USA) spectrophotometer. The evaluation activities for peroxidase (POD) by method of Doley and Jite (2014), superoxide dismutase (SOD) with Beauchamp and Fridovich (1971) procedure, catalase (CAT) using the technique of Krishnan and Murugan (2013), and polyphenol oxidase (PPO) were estimated by the Srivastava and Huystee (1973) methods. Caraway plant leaves were used as per each of the methods detailed in the supplementary material.

### 2.8 Yield parameters

At the harvesting stage in April 2024 and April 2025, the yield traits were recorded as follows: fruit yield/plant and the weight of 100 fruits.

#### 2.8.1 Fruits total proteins and total carbohydrates determination

The protein content of the seed was calculated by multiplying the percentage of the fruit samples mentioned before (2.12.2), whereas the nitrogen content in the seed was calculated by multiplying the percentage of the leaf samples by the factor 6.25. Total proteins (%) = N (%) x 6.25 (James, 1995), and total carbohydrates (%) were determined according to the method described by Herbert et al. (1971). Leaf pigments, leaf chemical composition, proline content, and enzyme assays (during the flowering stage) were determined in the second season only, and fruit total proteins and total carbohydrates were determined in the second season only.

### 2.9 Essential oil percentage and yield

Air-dried fruit materials at steady weight (10 g/sample of each replicate) were hydrodistilled with distilled water (0.5 L) in a Clevenger-type apparatus for three hours. The obtained essential oil (EO) was dried over anhydrous sodium sulfate and kept for later use at 4 °C (Elmsellem et al., 2019), where:


$$\text{EO }\%=\frac{\text{oil volume in graduated tub}}{\text{fruit sample weight }}\times 100$$

Plant EO yield (mL)= EO % x plant fruit weight.

### 2.10 GC- MS analysis

To find out the essential oil (EO) constituents during the 2^nd^ season, gas chromatography–mass spectrometry (GC–MS) was employed. Samples of each treatment for 3 repetitions were mixed carefully in one sample for GC-MS examination. First, the oil sample was filtered to prevent interference with the column, and 1 μL was injected into the GC system. An Agilent 6890N gas chromatograph fitted with an Agilent 5975B mass selective detector and a DB-5 MS capillary column (30 m × 0.25 mm × 0.25 μm; Agilent Technologies, USA) was used for the study. The injector operated in split mode at 250 °C. The oven temperature program was set as follows: an initial temperature of 60 °C (2 min held), then increased to 120 °C at 6 °C min^-^¹ (2 min held), and then to 230 °C at 4 °C min^-^¹ (5 min held). The split injection was performed using helium as the carrier gas at a flow rate of 1.0 mL min^-^¹. Mass spectra were recorded in electron ionization (EI) mode at 80 eV, with the transfer line at 280 °C and the ion source at 230 °C. The mass scan range covered m/z 30–1000. Indices of retention (IR) were recorded for a homologous series of n-alkanes (C_6_–C_26_) under the same conditions. Compound identification was achieved by comparing the mass spectra and IR spectra with those reported in NIST 08. L library of volatile oil compounds.

### 2.11 Statistical analysis

Data was analyzed using analysis of variance (ANOVA) for a split-plot design (Gomez and Gomez, 1984), with SAS software (Version 6.12; SAS Institute Inc., Cary, NC, USA). The main effects and their interactions were evaluated. Mean separations (± SE) were compared using Duncan’s Multiple Range Test (DMRT) at a significant level of *p* ≤ 0.05.

## 3 Results

### 3.1 Leaf relative water content, longest root length, and salinity tolerance index

Data in **Table 3** indicated that NaCl concentrations negatively affected LRWC%, LRL, and STI% compared with the control. The adverse effects of NaCl intensify with increasing concentration. Therefore, high LRWC (82.19 and 82.30%), LRL (26.28 and 27.37 cm), and STI (100 and 100%) were recorded for the control plants. On the opposite, the treatment of 4 gL^-^¹ NaCl had suggested the least LRWC (62.26 and 64.86%), LRL (21.08 and 21.32 cm), and STI (59.15 and 64.88%) in the two seasons, respectively. In the meantime, the findings suggested a beneficial effect of ZnO NP utilization on LRWC %, LRL, and STI % (**Table 3**) relative to the respective controls. So, ZnO NPs at 0.4 gL^-^¹ led to high LRWC (75.21 and 76.05%), LRL (24.50 and 25.76 cm), and STI (86.58 and 86.12%). While low LRWC (71.95 and 72.33%), LRL (22.83 and 22.88 cm), and STI (76.12 and 78.70%) were observed due to no spraying ZnO NPs in both seasons, respectively. In regard to the interaction between the two factors, distinctly, ZnO NPs at 0.4 gL^-^¹ under salt-free condition significantly elevated LRWC (86.23 and 86.45%), LRL (26.50 and 28.30 cm), and STI (100 and 100%) over the other treatments, regardless of the treatments of control and 0.2 gL^-^¹ ZnO NPs in case of STI under no salinity, which caused the same effect of 0.4 gL^-^¹ ZnO NPs on STI in the two seasons. Clearly, 4 gL^-^¹ NaCl without ZnO NP spraying resulted in significant reductions in LRWC (60.20 and 61.25%), LRL (19.37 and 19.20 cm), and STI (48.68 and 56.21%). The results suggested that the caraway plant exhibited good tolerance to salinity up to 4 g L^-^¹ NaCl following ZnO NPs at 0.4g/L^-1^ application. Generally, under salinity conditions, applying ZnO NPs at 0.2 gL^-^¹ or 0.4 gL^-^¹ improved LRWC %, LRL, and STI % in comparison to not applying ZnO NPs. It’s noted that yellowing and light chlorosis occur on the adult leaves during the growth seasons.

**Table 3:** Effect of NaCl, ZnO NPs and their interaction on relative water content, longest root length, and salinity tolerance index of caraway in 2023/2024 and 2024/2025 seasons.

Na Cl (g L^-1^)	ZnO NPs (g L^-1^)	RWC (%)		Longest root length (cm)		STI (%)	
		2023/2024	2024/2025	2023/2024	2024/2025	2023/2024	2024/2025
0 (Control)		82.19 ± 1.16a	82.30 ± 1.17a	26.28 ± 0.17a	27.37 ± 0.31a	100.00 ± 0.0a	100.00 ± 0.00a
1		78.60 ± 0.91b	78.82 ± 0.91b	24.42 ± 0.11b	25.82 ± 0.45b	88.63 ± 0.56b	90.97 ± 1.03b
2		73.31 ± 0.42c	73.75 ± 0.32c	23.76 ± 0.36c	24.37 ± 0.34c	84.55 ± 2.21c	82.67 ± 0.57c
3		71.08 ± 0.34d	71.52 ± 0.34d	23.01 ± 0.43d	22.61 ± 0.46d	80.01 ± 2.78d	72.45 ± 1.52d
4		62.26 ± 0.98e	64.86 ± 0.97e	21.08 ± 0.48e	21.32 ± 0.62e	59.17 ± 3.07e	64.88 ± 2.48e
	0.0 (Control)	71.95 ± 3.15c	72.33 ± 2.99c	22.83 ± 1.23c	22.88 ± 1.24c	76.12 ± 8.66c	78.70 ± 7.55c
	0.2	73.31 ± 4.02b	74.37 ± 2.78b	23.80 ± 0.74b	24.25 ± 1.13b	84.72 ± 6.89b	81.76 ± 6.49b
	0.4	75.21 ± 3.39a	76.05 ± 3.53a	24.50 ± 0.62a	25.76 ± 0.90a	86.58 ± 4.84a	86.12 ± 4.89a
0	0.0	78.33 ± 0.46c	78.55 ± 0.46c	26.5 ± 0.29a	26.37 ± 0.19c	100.00 ± 0.00a	100.00 ± 0.00a
	0.2	82.00 ± 0.23b	81.89 ± 0.36b	25.83 ± 0.17a	27.43 ± 0.23b	100.00 ± 0.00a	100.00 ± 0.00a
	0.4	86.23 ± 0.72a	86.45 ± 0.72a	26.5 ± 0.29a	28.3 ± 0.36a	100.00 ± 0.00a	100.00 ± 0.00a
1	0.0	75.67 ± 0.37d	75.89 ± 0.37d	24.4 ± 0.23bc	24.27 ± 0.15f	87.28 ± 0.19e	87.17 ± 0.87d
	0.2	81.8 ± 0.43b	78.55 ± 0.65c	24.37 ± 0.09bc	26.0 ± 0.25d	90.75 ± 0.83b	91.77 ± 0.64c
	0.4	78.33 ± 0.51c	82.02 ± 0.49b	24.5 ± 0.29b	27.2 ± 0.42b	87.87 ± 0.39de	93.97 ± 0.84b
2	0.0	74.17 ± 0.30e	74.39 ± 0.30e	22.5 ± 0.29d	23.33 ± 0.17g	75.74 ± 0.37g	81.46 ± 0.07f
	0.2	71.90 ± 0.54f	74.09 ± 0.38ef	24.03 ± 0.22bc	24.23 ± 0.15f	88.62 ± 0.87cd	81.65 ± 0.61f
	0.4	73.87 ± 0.35e	72.79 ± 0.31fg	24.73 ± 0.29bc	25.53 ± 0.29e	89.29 ± 0.38c	84.89 ± 0.46e
3	0.0	71.37 ± 0.74fg	71.59 ± 0.74g	21.37 ± 0.19e	21.23 ± 0.15i	68.89 ± 0.74h	68.64 ± 0.91j
	0.2	70.33 ± 0.31g	71.42 ± 0.74g	23.5 ± 0.15c	22.27 ± 0.18h	85.26 ± 0.27f	70.36 ± 0.55i
	0.4	71.53 ± 0.57fg	71.55 ± 0.21g	24.17 ± 0.20bc	24.33 ± 0.17f	85.87 ± 0.48f	78.35 ± 1.21g
4	0.0	60.20 ± 0.55i	61.25 ± 0.63j	19.37 ± 0.19f	19.20 ± 0.12j	48.68 ± 0.15j	56.21 ± 0.30l
	0.2	60.50 ± 0.58i	65.91 ± 0.49i	21.27 ± 0.15e	21.33 ± 0.17i	58.95 ± 0.65i	65.01 ± 0.56k
	0.4	66.07 ± 0.26h	67.42 ± 0.60h	22.6 ± 0.35d	23.43 ± 0.23g	69.88 ± 0.97h	73.41 ± 0.44h

Within a column, means with similar letters denote no significant difference (*P ≤* 0.05).

### 3.2 Leaf pigments

**Figure 2:**
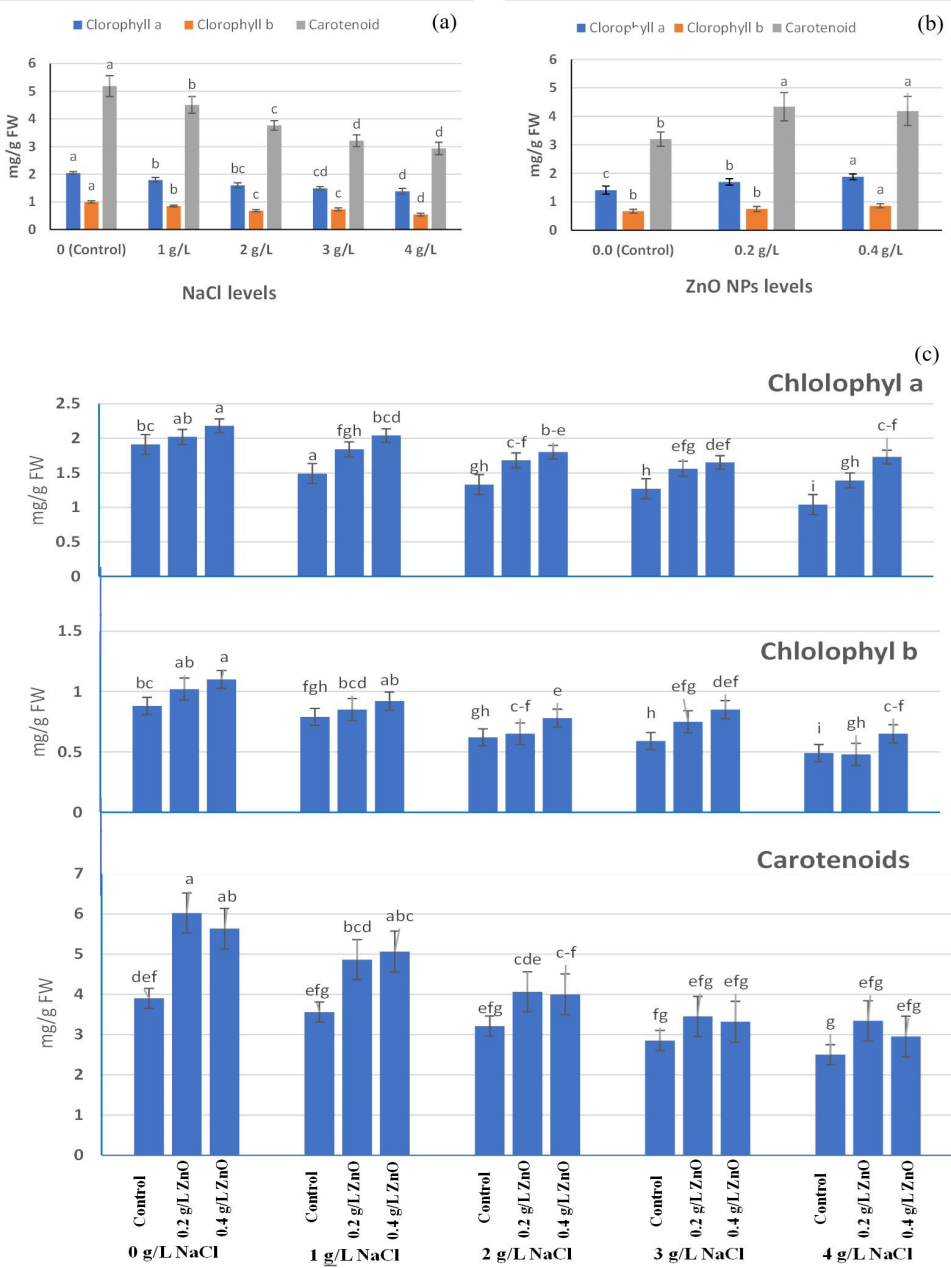
Effect of NaCl levels **(a)**, ZnO NPs levels **(b)** and their interaction **(c)** on chlorophyll a, chlorophyll b and carotenoid in the 2024/2025 season. Means having the same letters between the treatments denote non-significance (*P ≤* 0.05).

Chlorophyll a (Chl a), chlorophyll b (Chl b), and carotenoids (Cs) were significantly negatively affected by various NaCl concentrations, as shown in **Figure 2a**. The decrease in these traits was attributed to salinity. So, the highest significant Chl a (2.04 mg/g FW), Chl b (1.00 mg/g FW), and Cs (5.18 mg/g FW) were found in control plants. Conversely, 4 gL^-^¹NaCl caused the highest significant decrease in Chl a (1.38 mg/g FW), Chl b (0.54 mg/g FW), and Cs (2.91 mg/g FW). At the same time, the sprayed plants with 0.4 g L^-^¹ ZnO NPs showed significantly higher Chl a and Chl b of 1.88 and 0.86 mg/g FW, respectively. Spraying plants with 0.2 gL^-^¹ or 0.4 gL^-^¹ of ZnO NPs resulted in Cs contents of 4.34 and 4.19 mg/g FW, respectively, at the same significant level. On the opposite, the least Chl a, Chl b and Cs values of 1.41, 0.67, and 3.20 mg/g FW, in sequence, for the untreated plants (**Figure 2b**). In addition to the various combinations of NaCl and ZnO NPs exhibited significant impacts on leaf pigments (**Figure 2C**). Obviously, ZnO NPs at 0.2 and 0.4 gL^-^¹ under no salinity produced the highest significant values of Chl a and b and Cs without significant difference between the two treatments. Such treatments resulted in Chl a (2.02 and 2.18 mg/g FW), Chl b (1.02 and 1.10 mg/g FW), and Cs (6.02 and 5.63 mg/g FW). While lower significant values of Chl a and b and Cs were 1.04, 0.49 and 2.50 mg/g FW, respectively, due to irrigating plants with water containing 4 gL^-^¹ NaCl under no application of ZnO NPs. In general, across various NaCl levels, spraying plants with ZnO NPs at 0.2 gL^-^¹ or 0.4 gL^-^¹ increased leaf pigments more than non spraying ZnO NPs.

### 3.3 Leaf chemical composition

The results indicated that NaCl levels, ZnO NP levels, and their interaction significantly affected leaf chemical composition, namely proline content (PC) and percentages of N, P, K, Na, Cl, and Zn (**Table 4**). Moreover, N, P, and K %, and Zn concentration in the leaves of caraway decreased with increasing NaCl concentration, while PC and percentage of Na and Cl increased. Therefore, plants grown under non-salinity conditions had high concentrations of N (4.15%), P (0.46%), K (4.10%), and Zn (6.03 ppm). However, low significant values of N, P, K, and Zn were 2.77%, 0.32%, 2.36%, and 3.70 ppm, respectively, due to the application of 4 g L^-^¹ NaCl. In the meantime, 4 gL^-^¹ NaCl caused high PC (61.84 µmole/g FW), Na (0.68%), and Cl (1.05%), while the control plants contained low values of PC (43.59 µmole/g FW), Na (0.46%), and Cl (0.54%). The plant leaves that were sprayed with 0.4 g L^-^¹ ZnO NPs contained the highest significant N (3.67%), P (0.46%), K (3.62%), and Zn (4.81 ppm). Such plants had the lowest values for PC (47.02 µmole/g FW), Na (0.47%), and Cl (0.81%). In the meantime, the control plants contained high PC (63.02 µmole/g FW), Na (0.62%), and Cl (0.98%). Additionally, the control plants had low, non-significant N (2.98%), P (0.35%), K (2.82%), and Zn (4.00 ppm). The interaction data in **Table 4** indicate that leaf chemical composition was significantly affected by various combinations of NaCl and ZnO NPs. Whereas, at various NaCl and ZnO NP levels, particularly at 0.4 g L^-^¹, leaf chemical composition was improved by increasing N, P, K, and Zn, and by reducing PC, Na%, and Cl%. Furthermore, high contents of N, P, K and Zn were a result from application of 0.4 gL^-^¹ ZnO NPs under salt - free conditions. However, low values of these elements were observed in plants treated with 4 g L^-^¹ NaCl without ZnO NPs. In contrast, high values of PC, Na and Cl due to 4 gL^-^¹ NaCl under ZnO NPs - free condition. A low value of PC was observed for 0.0 ZnO NPs and no salinity and Na and Cl %, whereas a higher value of PC was observed for 0.4 gL^-^¹ ZnO NPs under no-salinity conditions.

**Table 4:** Effect of NaCl, ZnO NPs and their interaction on leaf chemical composition of caraway in the 2024/2025 season.

Na Cl (g L^-1^)	ZnO NP (g L^-1^)	Proline content (µmole/g FW)	N %	P %	K %	Na %	Cl %	Zn (ppm)
0 (Control)		43.59 ± 0.79 e	4.15 ± 0.03a	0.46 ± 0.04a	4.10 ± 0.07a	0.46 ± 0.01 e	0.54 ± 0.04e	6.03 ± 0.04 a
1		52.24 ± 2.31d	3.65 ± 0.09b	0.42 ± 0.03b	3.83 ± 0.11b	0.56 ± 0.02 d	0.91 ± 0.02d	4.53 ± 0.15 b
2		54.73 ± 2.93c	3.12 ± 0.19c	0.38 ± 0.01c	3.15 ± 0.19c	0.62 ± 0.01 c	0.96 ± 0.02c	4.27 ± 0.16 c
3		56.46 ± 3.02b	2.88 ± 0.17d	0.37 ± 0.02c	2.72 ± 0.13d	0.64 ± 0.00 b	1.00 ± 0.04b	4.00 ± 0.16 d
4		61.84 ± 4.43a	2.77 ± 0.12d	0.32 ± 0.01d	2.36 ± 0.16e	0.68 ± 0.01 a	1.05 ± 0.05a	3.70 ± 0.16 e
	0.0 (Control)	63.02 ± 6.18 a	2.98 ± 0.38c	0.35 ± 0.02b	2.82 ± 0.36c	0.62 ± 0.03 a	0.98 ± 0.13a	4.00 ± 0.51 c
	0.2	51.29 ± 1.97 b	3.31 ± 0.21b	0.36 ± 0.01b	3.25 ± 0.37b	0.60 ± 0.04 b	0.89 ± 0.05b	4.71 ± 0.36 b
	0.4	47.02 ± 0.94 c	3.67 ± 0.21a	0.46 ± 0.04a	3.62 ± 0.26a	0.57 ± 0.05 c	0.81 ± 0.09c	4.81 ± 0.36 a
0	0.0	41.37 ± 0.53j	4.12 ± 0.06a	0.40 ± 0.00cde	3.88 ± 0.11bc	0.51 ± 0.00h	0.49 ± 0.01i	5.94 ± 0.09 a
	0.2	44.53 ± 1.08ij	4.09 ± 0.01a	0.39 ± 0.01cde	4.15 ± 0.09ab	0.46 ± 0.00i	0.68 ± 0.02h	6.06 ± 0.03 a
	0.4	44.87 ± 1.02hij	4.24 ± 0.02a	0.60 ± 0.06a	4.25 ± 0.08a	0.42 ± 0.01j	0.45 ± 0.01i	6.10 ± 0.00 a
1	0.0	60.80 ± 0.60c	3.65 ± 0.02c	0.39 ± 0.00cde	3.45 ± 0.09d	0.60 ± 0.01f	0.98 ± 0.01cd	4.00 ± 0.00 e
	0.2	50.53 ± 0.50ef	3.34 ± 0.02de	0.37 ± 0.00def	3.88 ± 0.07bc	0.58 ± 0.03g	0.92 ± 0.01def	4.60 ± 0.10 c
	0.4	45.40 ± 1.21hi	3.96 ± 0.04ab	0.51 ± 0.05b	4.16 ± 0.04ab	0.51 ± 0.01h	0.84 ± 0.01 g	5.00 ± 0.00 b
2	0.0	65.87 ± 0.75b	2.45 ± 0.04h	0.36 ± 0.01efg	2.44 ± 0.03hi	0.65 ± 0.00cd	1.04 ± 0.05 c	3.65 ± 0.12 f
	0.2	51.87 ± 1.25ef	3.24 ± 0.15def	0.36 ± 0.01efg	3.39 ± 0.03de	0.62 ± 0.01e	0.92 ± 0.00def	4.60 ± 0.03 c
	0.4	46.47 ± 1.10ghi	3.68 ± 0.12bc	0.44 ± 0.01c	3.60 ± 0.23cd	0.59 ± 0.01fg	0.91 ± 0.01f	4.57 ± 0.05 c
3	0.0	67.97 ± 1.36b	2.24 ± 0.04h	0.32 ± 0.01fg	2.36 ± 0.22hi	0.65 ± 0.00cd	1.13 ± 0.10 b	3.37 ± 0.07 g
	0.2	52.93 ± 0.79e	3.01 ± 0.06fg	0.35 ± 0.00efg	2.72 ± 0.16gh	0.64 ± 0.01de	0.95 ± 0.01def	4.30 ± 0.08 d
	0.4	48.47 ± 1.45fgh	3.40 ± 0.09cd	0.43 ± 0.01cd	3.09 ± 0.05ef	0.64 ± 0.02de	0.92 ± 0.01 ef	4.32 ± 0.12d
4	0.0	79.07 ± 0.84a	2.42 ± 0.24h	0.30 ± 0.00g	1.98 ± 0.01j	0.70 ± 0.00a	1.24 ± 0.08 a	3.05 ± 0.03 h
	0.2	56.60 ± 0.14d	2.85 ± 0.04g	0.32 ± 0.01fg	2.13 ± 0.07ij	0.68 ± 0.02b	0.99 ± 0.01 cd	3.98 ± 0.07 e
	0.4	49.87 ± 1.03efg	3.05 ± 0.03efg	0.34 ± 0.00efg	2.98 ± 0.01fg	0.67 ± 0.01bc	0.93 ± 0.00def	4.06 0.03 e

Means that have the same letters throughout the column are not significantly different (*P ≤* 0.05).

### 3.4 Enzymatic activities

Catalase (CAT), peroxidase (POD), superoxide dismutase (SOD) and polyphenol oxidase (PPO) activities were notably affected by salinity stress (**Table 5**). At a high NaCl concentration, the activities of CAT, POD, SOD, and PPO reached their maximum. At this level, the activities were 19.14 mM H_2_O_2_ g^-^¹ FW min^-^¹ for CAT, 5.48 Unit-mg^-^¹ protein min^-^¹ for POD, 66.09 mM H_2_O_2_ g^-^¹ FW min^-^¹ for SOD and 0.53 Unit-mg^-^¹ protein min^-^¹ for PPO. While the least significant activities of such enzymes were observed in control plants, which reached 13.89 mM H_2_O_2_ g^-^¹ FW min^-^¹ for CAT, 4.37 Unit-mg^-^¹ protein min^-^¹ for POD, 31.63 mM H_2_O_2_ g^-^¹ FW min^-^¹ for SOD and 0.33 Unit-mg^-^¹ protein min^-^¹ for PPO. Additionally, ZnO NP spraying increased enzyme activities relative to the control (**Table 5**). Utilization of 0.2 gL^-^¹ ZnO NPs performed the maximum activities of CAT (17.83 mM H_2_O_2_ g^-^¹ FW min^-^¹), POD (5.25 Unit-mg^-^¹ protein min^-^¹), SOD (51.65 mM H_2_O_2_ g^-^¹ FW min^-^¹) and PPO (0.46 Unit-mg^-^¹ protein min^-^¹). This was followed by 0.4 g L^-^¹ ZnO NPs, then the control, which yielded the lowest enzyme activity. Additionally, the data in **Table 5** showed that applying ZnO NPs had a positive effect on enzyme activity across various NaCl concentrations. Therefore, the maximum activities of the mentioned enzymes were noticed under the combinations of ZnO NPs either at 0.2 gL^-^¹ or 0.4 gL^-^¹ with 4 gL^-^¹ NaCl. In contrast, the least significant activities of the aforementioned enzymes were observed under no ZnO NP spraying and salt-free utilization.

**Table 5:** ffect of NaCl, ZnO NPs and their interaction on antioxidant enzyme activities of caraway in the 2024/2025 season.

Na Cl (g L^-1^)	ZnO NPs (g L^-1^)	CAT (mM H_2_O_2_ g ^-1^ FWmin^-1^)	POD (unit-mg ^-1^ protein min ^-1^)	SOD (mM H_2_O_2_ g^-^¹ FW min^-^¹)	PPO (unit-mg ^-1^ protein min ^-1^)
0 (Control)		13.89 ± 0.70e	4.37 ± 0.20d	31.63 ± 2.26e	0.33 ± 0.02 e
1		16.00 ± 0.55d	4.71 ± 0.13c	49.98 ± 0.38d	0.43 ± 0.02 d
2		17.06 ± 0.45c	4.93 ± 0.11bc	51.99 ± 0.32c	0.46 ± 0.04 c
3		18.00 ± 0.41b	5.16 ± 0.08b	54.76 ± 0.57b	0.50 ± 0.03 b
4		19.14 ± 0.38a	5.48 ± 0.16a	66.09 ± 2.20a	0.53 ± 0.02 a
	0.0 (Control)	14.89 ± 1.14b	4.47 ± 0.26c	49.87 ± 3.16c	0.42 ± 0.03 b
	0.2	17.83 ± 0.87a	5.25 ± 0.14a	51.65 ± 5.77a	0.46 ± 0.04 a
	0.4	17.74 ± 0.69a	5.07 ± 0.19b	51.14 ± 7.85b	0.46 ± 0.04 a
0	0.0	11.13 ± 0.09i	3.60 ± 0.06i	38.60 ± 0.29h	0.31 ± 0.01i
	0.2	14.93 ± 0.30g	4.87 ± 0.12d-g	33.10 ± 0.23i	0.33 ± 0.01i
	0.4	15.60 ± 0.23f	4.63 ± 0.07fg	23.20 ± 0.21j	0.33 ± 0.01i
1	0.0	13.83 ± 0.15h	4.23 ± 0.07h	48.75 ± 0.61g	0.40 ± 0.00h
	0.2	17.20 ± 0.15c	5.10 ± 0.10b-e	50.35 ± 0.26f	0.44 ± 0.00fg
	0.4	16.97 ± 0.32d	4.80 ± 0.06efg	50.85 ± 0.32ef	0.45 ± 0.01ef
2	0.0	15.30 ± 0.12fg	4.57 ± 0.09g	51.25 ± 0.03ef	0.42 ± 0.01gh
	0.2	18.00 ± 0.00b	5.27 ± 0.09bc	51.52 ± 0.33e	0.49 ± 0.01bcd
	0.4	17.87 ± 0.32c	4.97 ± 0.03c-f	53.20 ± 0.06d	0.48 ± 0.01cd
3	0.0	16.40 ± 0.12e	4.93 ± 0.09c-f	53.37 ± 0.03d	0.47 ± 0.03de
	0.2	18.93 ± 0.30b	5.33 ± 0.09b	53.90 ± 0.23d	0.51 ± 0.01b
	0.4	18.67 ± 0.09b	5.20 ± 0.15bcd	57.00 ± 0.23 c	0.51 ± 0.01b
4	0.0	17.80 ± 0.25c	5.00 ± 0.12b-e	57.40 ± 0.52 c	0.50 ± 0.00bc
	0.2	20.07 ± 0.43 a	5.70 ± 0.06 a	69.40 ± 0.12 b	0.54 ± 0.01 a
	0.4	19.57 ± 0.20a	5.73 ± 0.32 a	71.47 ± 0.09 a	0.54 ± 0.01 a

Within a column, means with similar letters denote no significant difference (*P ≤* 0.05).

### 3.5 Fruit parameters

Application of NaCl, ZnO NPs levels, and their interaction significantly affected plant fruit yield and weight of 100 fruits of caraway (**Table 6**). So, high fruit yield was 8.48 and 8.71 g/plant for salt - free condition (control) against 5.23 and 5.29 g/plant for the treatment of 4 gL^-^¹ NaCl in the two seasons, respectively. Also, control plants yielded the heaviest 100-fruit weight of 1.18 g. In contrast, NaCl at 4 gL^-^¹ application produced the lightest significant weight of 100 fruits of 0.87 and 0.88 g in both seasons, consecutively. On the other hand, ZnO NPs foliar spraying at 0.4 gL^-^¹ produced the highest significant fruit yield (7.91 and 7.95 g/plant) and weight of 100 fruits (1.15 and 1.14 g) during the experimental seasons, respectively. In contrast, untreated plants with ZnO NPs yielded significantly lower fruit yields (5.39 and 6.13 g/plant) and 100-fruit weights (0.87 and 0.89 g) across both seasons. Obviously, the combinations of NaCl and ZnO NPs had different remarkable impacts on fruit traits (**Table 6**). The highest significant fruit yield (9.37 and 9.33 g/plant) and weight of 100 fruits (1.28 and 1.26 g) had been recorded for sprayed plants with 0.4 gL^-^¹ ZnO NPs under no salinity conditions. While the plants irrigated by water contained 4 gL^-^¹NaCl, without spraying by ZnO NPs resulted in a significantly lower fruit yield (3.36 and 4.00 g/plant) and the weight of 100 fruits (0.73 and 0.76 g) during the experimental seasons. The differences among the used combinations of NaCl and ZnO NPs were significant at the P ≤ 0.05 level in the majority of cases in the two seasons. Notably, ZnO NPs utilization enhanced fruit traits across different NaCl levels relative to no ZnO NP application.

**Table 6:** Effect of NaCl, ZnO NPs and their interaction on yield parameters of caraway in 2023/2024 and 2024/2025 seasons .

Na Cl (g L^-1^)	ZnO NPs (g L^-1^)	Fruits yield/plant (g)		Weight of 100 fruits (g)	
		2023/2024	2024/2025	2023/2024	2024/2025
0 (Control)		8.48 ± 0.29a	8.77 ± 0.26a	1.18 ± 0.03a	1.18 ± 0.03a
1		7.40 ± 0.32b	7.66 ± 0.23b	1.09 ± 0.05b	1.09 ± 0.05b
2		6.56 ± 0.41c	7.42 ± 0.25b	1.04 ± 0.06c	1.02 ± 0.05c
3		5.87 ± 0.51d	6.44 ± 0.37c	0.95 ± 0.05d	0.95 ± 0.04d
4		5.23 ± 0.44e	5.29 ± 0.41d	0.87 ± 0.04e	0.88 ± 0.04e
	0.0 (Control)	5.39 ± 0.71c	6.13 ± 0.70c	0.87 ± 0.07c	0.89 ± 0.06c
	0.2	6.82 ± 0.61b	7.26 ± 0.64b	1.06 ± 0.05b	1.05 ± 0.06b
	0.4	7.91 ± 0.43a	7.95 ± 0.43a	1.15 ± 0.06a	1.14 ± 0.05a
0	0.0	7.73 ± 0.12bc	8.03 ± 0.15b	1.10 ± 0.07c	1.09 ± 0.09bc
	0.2	8.33 ± 0.33b	8.93 ± 0.44a	1.16 ± 0.04bc	1.20 ± 0.04a
	0.4	9.37 ± 0.44a	9.33 ± 0.29a	1.28 ± 0.03a	1.26 ± 0.04a
1	0.0	6.20 ± 0.20e	6.90 ± 0.09cd	0.92 ± 0.02de	0.90 ± 0.015ef
	0.2	7.79 ± 0.12bc	7.97 ± 0.41b	1.13 ± 0.04c	1.16 ± 0.04ab
	0.4	8.20 ± 0.20b	8.10 ± 0.10b	1.23 ± 0.03ab	1.22 ± 0.03a
2	0.0	5.00 ± 0.12f	6.56 ± 0.20d	0.83 ± 0.04ef	0.86 ± 0.02fg
	0.2	6.94 ± 0.06d	7.65 ± 0.38bc	1.10 ± 0.06c	1.00 ± 0.01cde
	0.4	7.74 ± 0.12bc	8.04 ± 0.08b	1.18 ± 0.02bc	1.20 ± 0.03a
3	0.0	4.08 ± 0.57g	5.18 ± 0.13e	0.78 ± 0.02fg	0.83 ± 0.02fg
	0.2	6.19 ± 0.11e	6.52 ± 0.29d	0.97 ± 0.05d	0.97 ± 0.05de
	0.4	7.34 ± 0.16cd	7.62 ± 0.17bc	1.10 ± 0.00c	1.05 ± 0.05cd
4	0.0	3.96 ± 0.11g	4.00 ± 0.33f	0.73 ± 0.06g	0.76 ± 0.08g
	0.2	4.86 ± 0.21f	5.22 ± 0.21e	0.93 ± 0.05d	0.91 ± 0.03ef
	0.4	6.88 ± 0.18d	6.99 ± 0.01cd	0.96 ± 0.07d	0.98 ± 0.04de

Within a column, means with similar letters denote non-significance (*P ≤* 0.05).

### 3.6 Fruits total proteins and total carbohydrates

**Figure 3:**
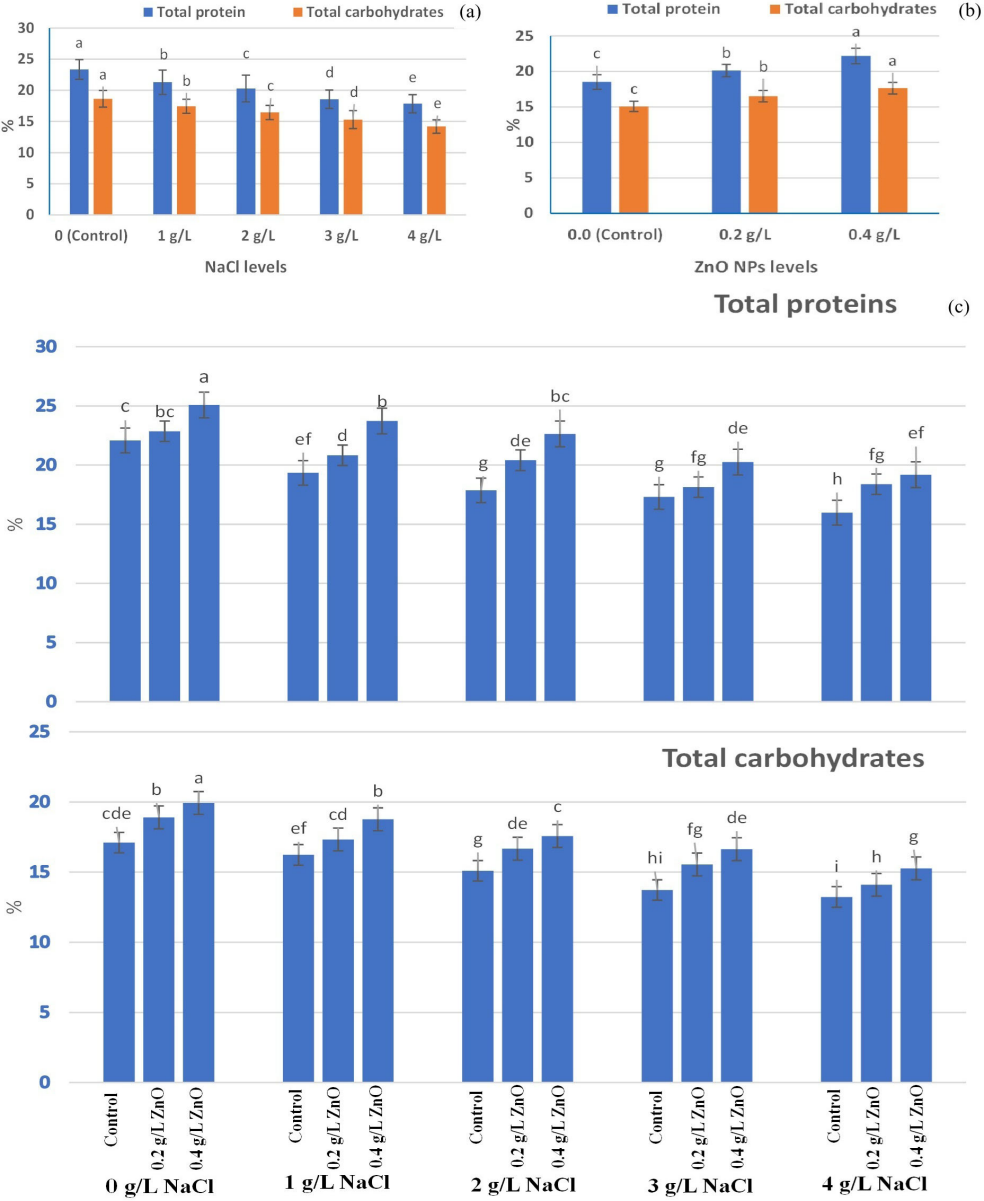
Effect of NaCl levels **(a)**, ZnO NPs levels **(b)** and their interaction **(c)** on total proteins and total carbohydrates in the 2024/2025 season.Means with the same letters within the figure denote non-significance (*P ≤* 0.05).

Data illustrated in **Figure 3** (a, b and c) show the effects of the used levels of NaCl, ZnO NPs, and their interaction on total proteins (TP) and total carbohydrates (TC), in fruits of caraway. In relation to control, increasing NaCl led to a significant decrease in TP and TC % (**Figure 3a**). High TP and TC in fruits were 23.34 and 18.64%, respectively, for control treatment. However, NaCl at 4 g L^-^¹ reduced TP and TC to 17.85% and 14.20%, respectively. The differences among the NaCl levels were significant (*P* ≤ 0.05). As well as the application of ZnO NPs, TP and TC % were enhanced relative to the respective controls (**Figure 3b**). High TP (22.18%) and TC (17.64%) referred to 0.4 gL^-^¹ ZnO NPs, followed by 20.12 and 16.51% for TP and TC, respectively. In the 0.2 g L^-^¹ ZnO NP control treatment, TP and TC were 18.51% and 15.07%, respectively. The differences among the ZnO NPs treatments were significant (*P* ≤ 0.05). Also, it was noticed that the various combinations of NaCl and ZnO NPs significantly affected TP and TC % in caraway fruits (**Figure 3c**). Clearly, under the salinity levels used, the utilization of ZnO NPs improved these traits compared with no ZnO NPs spraying. Therefore, 0.4 gL^-^¹ ZnO NPs under no salinity realized high TP (25.08%) and TC (19.93%), on the opposite, low TP (15.98%) and TC (13.23%) were found in plant fruits receiving 4 gL^-^¹ NaCl without ZnO NPs application. The differences among the combinations of NaCl and ZnO NPs were significant (P ≤ 0.05) in most cases for TP and TC%.

### 3.7 Essential oil productivity

The percentage of total oil (EO %) and essential oil yield (EOY) were negatively affected under salinity stress relative to no salinity during the two seasons (**Table 7**). The negative effect increased by increasing NaCl level. The control plants yielded the highest significant EO% (5.81 and 5.61%) and EOY (0.53 and 0.50 mL/plant) across the two seasons, respectively. Conversely, NaCl at 4 gL^-^¹ treatment produced the least significant EO % (3.47 and 3.36%) and EOY (0.18 and 0.18 mL/plant). Distinctly, the plants sprayed by 0.4 gL^-^¹ produced fruits with highly significant EO% (5.20 and 4.93%) and EOY (0.43 and 0.40 mL/plant). Followed by 0.2 gL^-^¹ ZnO NPs, which gave EO% of 4.43 and 4.28% and EOY of 0.32 and 0.32 mL/plant. Ultimately, the control plants produced EO% of 3.82 and 3.36, and EOY of 0.23 and 0.23 mL/plant in the two seasons, respectively (**Table 7**). Under salinity and no-salinity conditions, the application of ZnO NPs increased EO% and EOY relative to the no-application control (**Table 7**). Thus, the maximum significant EO% (6.92 and 6.72%) and EOY (0.68 and 0.63 mL/plant) were found in the plant fruits sprayed by 0.4 gL^-^¹ ZnO NPs grown under no salinity stress in the two seasons. Conversely, the minimum EO% (3.25 and 3.05%) and EOY (0.13 and 0.12 mL/plant) were produced from plants grown under 4 gL^-^¹ NaCl without applying of ZnO NPs in the two seasons.

**Table 7:** Effect of NaCl, ZnO NPs and their interaction on essential oil percentage and yield per plant of caraway in 2023/2024 and 2024/2025 seasons.

Na Cl (g L^-1^)	ZnO NP (g L^-1^)	Essential oil %		Essential oil yield (mL) per plant	
		2023/2024	2024/2025	2023/2024	2024/2025
0 (Control)		5.81 ± 0.35a	5.61 ± 0.35a	0.53 ± 0.04a	0.50 ± 0.04a
1		4.89 ± 0.23b	4.66 ± 0.21b	0.39 ± 0.03b	0.36 ± 0.02b
2		4.31 ± 0.22c	4.05 ± 0.19c	0.30 ± 0.03c	0.30 ± 0.02c
3		3.94 ± 0.21d	3.74 ± 0.18d	0.24 ± 0.03d	0.25 ± 0.02d
4		3.47 ± 0.15e	3.36 ± 0.22e	0.18 ± 0.02e	0.18 ± 0.02e
	0.0 (Control)	3.82 ± 0.28c	3.63 ± 0.27c	0.23 ± 0.05 c	0.23 ± 0.04c
	0.2	4.43 ± 0.41b	4.28 ± 0.41b	0.32 ± 0.06 b	0.32 ± 0.06b
	0.4	5.20 ± 0.53a	4.93 ± 0.51a	0.43 ± 0.07 a	0.40 ± 0.06a
0	0.0	4.67 ± 0.33def	4.47 ± 0.33de	0.39 ± 0.01c	0.36 ± 0.03cd
	0.2	5.83 ± 0.22b	5.63 ± 0.22b	0.51 ± 0.01b	0.50 ± 0.04b
	0.4	6.92 ± 0.08a	6.72 ± 0.08a	0.68 ± 0.01a	0.63 ± 0.03a
1	0.0	4.25 ± 0.25efg	4.05 ± 0.25def	0.29 ± 0.01ef	0.28 ± 0.02efg
	0.2	4.75 ± 0.14de	4.62 ± 0.20cd	0.38 ± 0.01c	0.37 ± 0.03cd
	0.4	5.67 ± 0.22bc	5.30 ± 0.14bc	0.48 ± 0.01b	0.43 ± 0.02c
2	0.0	3.67 ± 0.08ghi	3.47 ± 0.08fg	0.18 ± 0.00h	0.23 ± 0.01gh
	0.2	4.17 ± 0.17efg	4.13 ± 0.33def	0.30 ± 0.01e	0.32 ± 0.04def
	0.4	5.08 ± 0.22cd	4.55 ± 0.14de	0.41 ± 0.02c	0.37 ± 0.01cd
3	0.0	3.25 ± 0.00i	3.13 ± 0.08g	0.15 ± 0.01hi	0.16 ± 0.01hi
	0.2	4.00 ± 0.14fgh	3.80 ± 0.14efg	0.25 ± 0.01fg	0.25 ± 0.01g
	0.4	4.58 ± 0.22def	4.30 ± 0.14de	0.34 ± 0.02d	0.33 ± 0.00de
4	0.0	3.25 ± 0.14i	3.05 ± 0.14g	0.13 ± 0.01i	0.12 ± 0.01i
	0.2	3.42 ± 0.22hi	3.22 ± 0.22g	0.17 ± 0.02hi	0.17 ± 0.02hi
	0.4	3.75 ± 0.38ghi	3.80 ± 0.58efg	0.26 ± 0.02fg	0.25 ± 0.03g

Within a column, means with similar letters denote no significant difference (*P ≤* 0.05).

### 3.8 Essential oil analysis

GC–MS analysis of caraway EO (**Table 8**) identified 32 constituents across 15 applications. The maximum EO compounds are 14, resulting from T1, while T12 and T14 resulted in the least EO compounds of the three compounds. Total compounds accounted for 100% across all treatments. Monoterpene hydrocarbons ranged from 43.63% in T9 to 64.63% in T4. Sesquiterpene hydrocarbons ranged from 0.00% in 13 treatments to 2.12% and 6.03% in T4 and T1, respectively. While oxygenated hydrocarbons reached 32.25% in T4 to 56.37% in T9. Only Carvone was found in the EO of all treatments, followed by Limonene, which was identified in the EO of all treatments except T8; D-Limonene was identified in the EO of all treatments except T9 and T10. While some compounds as numbers of 6,7,9,10,11, and 12 were observed in EO of T1 only, numbers 17,18,19 and 20, were noted in T2 only, numbers 21,22,23,24 and 25 were identified in T4 only, number 26 was in T5 only, number 28 was in T8 only, numbers 29 and 30 were found to be in T9 only and numbers 31 and 32 were to be found in T10. The main compounds were carvone (49.71% in T8), limonene (28.68% in T9), D-limonene (25.95% in T13), and camphene (16.75% in T8). The data indicate that combinations of NaCl and ZnO NPs have differently effects on EO composition.

**Table 8:** Effect of the different combinations of NaCl and ZnO NPs on caraway essential oil analysis in 2024/2025.

Compound name (%)		Treatments																		
		T1	T2	T3	T4	T5	T6	T7	T8	T9	T10	T11	T12		T13		T14		T15	
1.	Carvone	32.02	40.59	37.65	33.25	39.08	37.09	38.09	49.71	41.69	39.75	39.68	48.08		40.97		46.75		42.57	
2.	Limonene	23.9	21.06	25.03	14.21	22.85	22.08	27.36	–	28.68	24.68	26.75	27.56		23.63		28.6		24.96	
3.	D-Limonene	21.11	14.06	24.88	10.08	24.75	19.23	24.89	24.85	–	–	24.62	24.36		25.95		24.65		23.85	
4.	á-Pinene	1.23	5.12	–	–	–	–	–	–	–	9.60	–	–		–		–		–	
5.	Camphene	0.85	–	–	–	–	–	–	16.75	14.95	14.65	–	–		–		–		–	
6.	Cyclopropane, 1,1-dimethyl-2-(2,4-pentadienyl)-	0.18	–	–	–	–	–	–	–	–	–	–	–		–		–		–	
7.	3-Methyl-6-hepten-1-yn-3-ol	0.09	–	–	–	–	–	–	–	–	–	–	–		–		–		–	
8.	Terpinyl acetate	4.32	–	7.36	–	5.08	–	–	–	–	–	8.95	–		–		–		8.62	
9.	Borneol	4.06	–	–	–	–	–	–	–	–	–	–	–		–		–		–	
10.	Thujol	3.02	–	–	–	–	–	–	–	–	–	–	–		–		–		–	
11.	1,5,7-Octatrien-3-ol, 2,6-dimethyl-	1.72	–	–	–	–	–	–	–	–	–	–	–		–		–		–	
12	6-Methyl-3,5,8,8a-tetrahydro-1H-2-benzopyran	4.02	–	–	–	–	–	–	–	–	–	–	–		–		–		–	
13.	1H-2-Benzopyran, 3,5,8,8a-tetrahydro-6-methyl-	2.01	–	–	–	–	4.36	–	–	–	–	–	–		–		–		–	
14.	Myrtenal	1.47	3.05	–	–	–	7.49	–	–	–	–	–	–		–		–		–	
15.	γ-Terpinene	–	3.08	5.08	–	–	–	–	–	–	–	–	–		–		–		–	
16.	Spiro [2.4] heptane, 1,5-dimethyl-6-methylene-	–	2.81	–	2.65	–	–	–	–	–	–	–	–		–		–		–	
17.	Cyclohexanone, 2-methyl-5-(1-methylethenyl)-, trans-	–	1.98	–	–	–	–	–	–	–	–	–	–		–		–		–	
18.	Cyclohexanone, 2-methyl-5-(1-methylethenyl)-	–	1.44	–	–	–	–	–	–	–	–	–	–		–		–		–	
19.	Tricyclo[3.3.0.0(2,8)] octan-3-one, 5,8-dimethyl-	–	4.35	–	–	–	–	–	–	–	–	–	–		–		–		–	
20.	Terpenoid alcohol	–	2.46	–	–	–	–	–	–	–	–	–	–		–		–		–	
21.	Myrcene	–	–	–	11.08	–	–	–	–	–	–	–	–		–		–		–	
22.	β-Phellandrene	–	–	–	14.8	–	–	–	–	–	–	–	–		–		–		–	
23.	β-Ocimene	–	–	–	9.56	–	–	–	–	–	–	–	–		–		–		–	
24.	β-Thujene Spiro isomer	–	–	–	2.25	–	–	–	–	–	–	–	–		–		–		–	
25.	α-Farnesene	–	–	–	2.12	–	–	–	–	–	–	–	–		–		–		–	
26.	Carene	–	–	–	–	1.24	–	–	–	–	–	–	–		–		–		–	
27.	p-Cymene	–	–	–	–	7.00	9.75	9.66	–	–	–	–	–		9.45		–		–	
28.	Bicyclo[4.1.0] heptane, 7-(1-methylethylidene)-	–	–	–	–	–	–	–	8.69	–	–	–	–		–		–		–	
29.	Cyclohexene, 5-methyl-3-(1-methylethenyl)-, trans-(-)	–	–	–	–	–	–	–	–	6.06	–	–	–		–		–		–	
30.	Cyclohexene, 4-methyl-1-(1-methylethenyl)-	–	–	–	–	–	–	–	–	8.62	–	–	–		–		–		–	
31.	α-Camphene	–	–	–	–	–	–	–	–	–	8.06	–	–		–		–		–	
32.	Bornane	–	–	–	–	–	–	–	–	–	3.26	–	–		–		–		–	
	Total Compounds (%)	100	100	100	100	100	100	100	100	100	100	100	100		100		100		100	
	Monoterpene Hydrocarbons (%)	47.27	46.13	54.99	64.63	55.84	51.06	61.91	50.29	43.63	60.25	51.37	51.92		59.03		53.25		48.81	
	Sesquiterpene Hydrocarbons (%)	6.03	–	–	2.12	–	–	–	–	–	–	–	–		–		–		–	
	Oxygenated Hydrocarbons (%)	46.7	53.87	45.01	33.25	44.16	48.94	38.09	49.71	56.37	39.75	48.63	48.08		40.97		46.75		51.19	
	Number of Compounds (%)	14	11	5	9	6	6	4	4	5	6	4	3		4		3		4	

T1–0 gL^-1^ NaCl + 0 gL^-1^ ZnO NPs (control), T2–0 gL^-1^ NaCl + 0.2 gL^-1^ ZnO NPs, T3–0 gL^-1^ NaCl + 0.4 gL^-1^ ZnO NPs, T4–1 g/L NaCl + 0 g/L ZnO NPs, T5–1 gL^-1^ NaCl + 0.2 gL^-1^ ZnO NPs, T6–1 gL^-1^ NaCl + 0.4 gL^-1^ ZnO NPs, T7–2 gL^-1^ NaCl + 0 gL^-1^ ZnO NPs, T8–2 g/L NaCl + 0.2 g/L ZnO NPs, T9–2 g/L NaCl + 0.4 gL^-1^ ZnO NPs, T10- gL^-1^ NaCl + 0 gL^-1^ ZnO NPs, T11–3 gL^-1^ NaCl + 0.2 gL^-1^ ZnO NPs, T12–3 gL^-1^ NaCl + 0.4 g/L ZnO NPs, T13–4 g/L NaCl + 0 g/L ZnO NPs, T14–4 gL^-1^ NaCl + 0.2 gL^-1^ ZnO NPs and T15–4 gL^-1^ NaCl + 0.4 gL^-1^ ZnO NPs.

## 4 Discussion

Salt stress affects biochemical and physiological processes at all stages of the plant life cycle. It is also a major abiotic stress that drastically affects plant development, causing osmotic stress and inducing imbalances in nutrient and ionic levels. These imbalances negatively affect plant growth and development through a range of physiological and biochemical mechanisms (Zhang et al., 2013). By increasing Na and Cl ion levels in plant cells, salt stress inhibits morphological, physiological, epigenetic, and genetic characters, leading to increased production of oxidative stress (Weng et al., 2025) and reduced uptake of nutrients and water, which in turn significantly decreases plant growth. Moreover, salinity stress increases electrolyte leakage and malondialdehyde (MDA) accumulation and decreases RWC in plants by disrupting water uptake (Nassar et al., 2020).

A reduction was observed in yield traits, chemical and biochemical composition, and essential oil productivity of caraway plants under salinity, which may be due to ion toxicity and osmotic stress, leading to nutrient imbalance and oxidative stress. This is attributed to increased Na^+^ and Cl^-^ uptake (Zhu et al., 2016). Also, a greater decrease in biomass was observed in sensitive coriander cultivars than in resistant cultivars under various NaCl applications (Meriem et al., 2014). Salinity increases osmotic stress, which reduces water uptake and transport. This decreases triggers hormone-induced sequential reactions that lead to reduced stomatal opening, CO_2_ assimilation, and photosynthetic rates (Sarker and Oba, 2020).

The growth reduction might also be due to the energy required to divert from growth to salt-stress homeostasis and a decrease in carbon gain (Sarkar and Oba, 2020). Moreover, NaCl induces nutrient imbalance, increased ROS generation, and reduced enzyme activity, which can damage cellular components and biological membranes, thereby reducing growth (Alzahrani et al., 2019). It inhibits cellular and metabolic processes under osmotic stress, disrupts enzyme dynamics, and interferes with plasma membrane function (Hussain et al., 2021). It also decreases water availability due to the high soil osmotic potential (Soltabayeva et al., 2021). Our data are in accordance with the results of Naghavi et al. (2024), who found that the number of seeds/plant and umbrellas, the weight of 1000 seeds, and seed yield/plant of caraway were significantly reduced under irrigation water with 3 and 5 dS/m NaCl, with the maximum reduction at high salinity. According to Yolcu and Yilmaz (2025), NaCl at 50 and 100 mM negatively affected herb weight and root length of sweet basil. Akbari Ghogdi et al. (2012) reported that high NaCl levels affect photosynthesis, and long-term exposure to salt stress reduces the synthesis of the chlorophyll protein-lipid complex. Also, under NaCl at 6 dS/m, Chl a, Chl b, and carotenes in *O. basilicum* and *O. minimum* significantly decreased, and the reduction of these traits was higher in *O.minimum* than in *O. basilicum* (Heidari, 2012). Hassan (2019) indicated that Chl a, Chl b, and carotenes in caraway leaves under irrigation water with a salinity of 2500 ppm were lower than those under 250–1750 ppm. However, Ghorbani et al. (2018) revealed that RWC %, Chl a, Chl b, total Chl, and carotenoids in *Mentha piperita* were highest in the control plants, and the values of these parameters were significantly lowest under 6 dS/m². Furthermore, Estaji et al. (2018) demonstrated that chlorophyll (Chl) levels in plants are a reliable indicator of photosynthetic activity under stress conditions because they play a vital role in estimating the photosynthetic rate. Yaichi et al. (2025) concluded that NaCl at 50 and 100 mM decreased photosynthetic pigments, SPAD units, chlorophyll fluorescence data (Fm, Fv, Fv/Fm), and carotenoid contents in *Dracocephalum moldavica* plants relative to their respective controls. Morshedloo et al. (2025) found a reduction in chlorophyll level in *Mentha × gracilis* under NaCl at 50 and 100 mM. Jamil et al. (2007) found that resistance of photosystem II (PSII) to high salinity pressure and an increase in chlorophyll level played an essential role in the salinity resistance of sugar beet and cabbage, suggesting that PSII may play a vital function in the salinity stress tolerance of water dropwort (Kumar et al., 2021).

Under various NaCl levels, leaf N, P, K %, and Zn content decreased, while Na %, Cl %, and proline content in caraway leaves increased, potentially due to the increased osmotic potential of the soil solution resulting from saline irrigation water. Increased Na and Cl in the soil adversely affect the uptake of essential nutrients such as N, P, K, Zn, Mg, Ca, Mn, Cu, Fe, and water by plant roots, subsequently reducing the vital nutrient and water content in plant cells (Ismail et al., 2014). This can reduce the function of essential nutrients in the synthesis of important compounds and energy, as N, P, K, and Zn are employed in the synthesis of carbohydrates, amino acids, nucleic acids, proteins, lipids, and energy compounds, and serve as co-enzymes in physiological processes in plant cells (Hawkesford et al., 2012). Therefore, the reductions in fruit yield, 100-fruit weight, TP and TC, EO%, and EO yield may be attributed to decreases in leaf nutrients and RWC% and to increases in Na% and Cl% in plant cells under salinity stress. Xue et al. (2021) reported that increased soil salinity negatively affects plant metabolism and reduces cellular membrane integrity, nutritional balance, and redox homeostasis, thereby altering stomatal activity and inducing changes in secondary metabolites. Kasrati et al. (2014) found that NaCl at 100 and 150 mM decreased EO content and quality, K %, and K/Na ratio, and increased Na^+^ content in *Mentha sauveolens*. Chrysargyris et al. (2018) reported that NaCl at 100 mM decreased EO yield, phenolic content, and antioxidant status in *Lavandula angustifolia*. Additionally, the increase in Na^+^ accumulation in the cytosol is related to salt-induced K^+^ efflux, and the cytosolic ratio of K^+^/Na^+^ strongly reduces under salinity conditions (Sarker and Oba, 2020). This may be related to the passive entry of Na^+^ into the roots via voltage-independent or weakly voltage-independent nonselective channels, or via other Na^+^ transporters, such as high-affinity K^+^ transporter members. Furthermore, the sensitivity of plant species to salinity depends on their capacity for uptake and dispersal of Na^+^ between the roots and shoots (Evelin et al., 2012).

The displacement of K^+^ by Na^+^ in enzyme-binding sites inhibits cellular functions (Wu, 2018). Tolerant cultivars under stress conditions can regulate osmotic pressure by accumulating various compounds such as proline, soluble sugars, and proteins, and by increasing the activity of antioxidants such as SOD, POD, CAT, and PPO, which help plants cope with salinity stress (Sarker and Oba, 2019). Proline and soluble sugars protect plant cells under stress by maintaining the cytosolic osmotic potential relative to the vacuolar osmotic potential and the external environment. Proline is also utilized to combat ROS, in addition to protecting enzymes and stabilizing their structures (Alzahrani et al., 2019). Salinity-tolerant species, for instance *Mentha* × *gracilis* (Morshedloo et al., 2025) and *O. basilicum* (Shabani et al., 2025), show increased proline content.

In salt-sensitive cultivars, proline content decreased following exposure to 100 mM NaCl (Chun et al., 2018). The decrease in proline under 200 mM NaCl stress may be attributed to reduced activity of proline biosynthesis pathway enzymes (P5CS and glutamine dehydrogenase) in the VIIEO 135 cultivar of water dropwort (Chun et al., 2018). Proline dehydrogenase (ProDH), a key enzyme regulating proline accumulation, may also contribute. Consequently, ProDH1 and ProDH2 gene expression may be reduced under higher NaCl concentrations (Chun et al., 2018; Kumar et al., 2021).

In the current study on caraway, CAT, SOD, POD, and PPO activities increased relative to their respective controls as NaCl concentration increased, with peak activity observed at 4 g L^-1^ NaCl. This observation aligns with Polash et al. (2019), who reported that elevated enzyme activity enhances salt stress tolerance by scavenging ROS, and that resistant species exhibit higher enzyme activities than sensitive species. SOD serves as the primary line of defense, converting superoxide into H_2_O_2_, which is then processed by CAT into H_2_O and O_2_. POD further contributes to H_2_O_2_ removal, particularly within the chloroplast (Polash et al., 2019). Similarly, PPO, a terminal oxidase, facilitates direct electron transport to O_2_ via plant respiration, involving the oxidation of intermediate products. PPO catalyzes the conversion of phenols to quinones and participates in the synthesis of phenol-containing cell wall substances, such as lignin. Polyphenol oxidase activity increases under stress conditions, aiding plant detoxification by converting phenolic compounds into less reactive forms, thereby reducing ROS accumulation (Rivero et al., 2001). Increased SOD, CAT, POD, and PPO activities under salinity have been previously reported in water dropwort (Kumar et al., 2021), *Metha* x *gracilis* (Morshedloo et al., 2025), and *Glycyrrhiza uaralensis* (Wu and Ma, 2025). Thus, NaCl adversely affects caraway fruit quality, as indicated by decreases in TP, TC, and EO%.

Conversely, our results clearly show that applying ZnO NPs enhances the measured traits compared with non-application. This enhancement may be attributed to the crucial role of ZnO NPs in physiological, chemical, and biochemical functions within plant cells (Marschner, 2012). ZnO NPs exhibit high uptake and transport, ensuring adequate Zn availability throughout plant life stages and development. Zinc can stabilize the cell membrane and enhance cell division and elongation, thereby increasing fresh and dry weights (Elsilk et al., 2024). Furthermore, Zn plays a principal role in activating numerous enzymes (approximately 300) involved in protein synthesis, carbohydrate metabolism, pollen structure formation, and other key physiological processes in plants (Sadeghzadeh and Rengel, 2011). Additionally, ZnO NPs are used as adsorbents, stimulants, polymer additives, and antibacterial agents owing to their large specific surface area, high pore volume, long lifespan, photodegradability, and low toxicity (Abbasi Khalaki et al., 2021). Thus, Zn is essential for chlorophyll synthesis and growth and development, DNA synthesis, RNA metabolism, and pollen function (Cakmak, 2008). The use of ZnO NPs has been shown to enhance growth traits and leaf pigments in *Glycyrrhiza uralensis* (Wu and Ma, 2025), rosemary (Al-Khazraji et al., 2025), and *Dracocephalum moldavica* (Yaichi et al., 2025).

The effects of ZnO NPs depend on the rate, plant species, and the timing of application (Nemati Lafmejani et al., 2021). Maximum values for growth parameters and photosynthetic pigments in valerian, chicory, purple coneflower, and Withania were observed following spraying with 3 g L^-1^ ZnO NPs at 20, 40, and 60 days post-transplanting (Rabary et al., 2022). Similarly, 300 mg L^-1^ ZnO NPs significantly increased carotenoid levels in Arabidopsis plants by upregulating the expression of various carotenoid biosynthesis genes (Wang et al., 2016). Yaichi et al. (2025) reported that 1 g L^-1^ ZnO NPs increased carotenoid content in *Dracocephalum moldavica* compared with the untreated control. Our data show that spraying ZnO NPs increased leaf concentrations of essential elements (N, P, K, Zn) and RWC, whereas decreasing leaf Na, Cl, and proline levels relative to controls. Following foliar application of ZnO NPs, Zn can accumulate in plant leaves and serve as an active Zn source for plant metabolism (Li et al., 2019). Rosemary Zn content increased after foliar application of 3 mg L^-1^ ZnO NPs (Hassanpouraghdam et al., 2020). Mehrabani et al. (2018) demonstrated that 10 mg L^-1^ ZnO NPs boosted Zn and reduced Na levels, while 5 mg L^-1^ ZnO NPs raised K content in rosemary leaves compared with the respective control. Nano zinc at 1 mg L^-1^ induced the highest N, P, and Zn contents in barley grains (Bachai and Jamil, 2025). Likewise, ZnO NPs at 1 g L^-1^ increased K content and decreased Na content in the leaves of *Dracocephalum moldavica* (Yaichi et al., 2025). Previously, El-Agouze et al. (2022) reported that ZnO NPs at 0.25 – 1.00 g L^-1^increased leaf N, P, and K contents and fruit Zn content in *Silybum marianum* plants relative to untreated plants.

Enhanced antioxidant enzyme activity, including catalase (CAT), superoxide dismutase (SOD), peroxidase (POD), and polyphenol oxidase (PPO), were observed in caraway leaves treated with zinc oxide nanoparticles (ZnO NPs). Similarly, ZnO NPs have been shown to increase CAT, SOD, and POD activity in *Glycyrrhiza uralensis* (Wu and Ma, 2025). In sweet violet, the most effective treatments for increasing POD, ascorbate peroxidase (APX), and relative water content (RWC) were 1000 and 1500 mg L^-1^ ZnO NPs at 55% field capacity, respectively; however, control plants exhibited higher proline content (Bagheri et al., 2021). The stimulatory effects of ZnO NPs on the chemical composition of leaves, photosynthetic pigments, RWC, and the activities of POD, SOD, CAT, and PPO enzymes in caraway resulted in enhanced fruit yield, TP, TC, essential oil productivity (EO% and EOY), and stress tolerance index (STI). Zinc plays a crucial role in carbohydrate synthesis, protein metabolism, and auxin regulation, thereby affecting EO levels (Boradley et al., 2007). Spraying caraway plants with 50 and 100 mg L^-1^ ZnO NPs significantly increased EO% in both seasons and EOY and fruit yield per plant in the second season (El-Sayed et al., 2007). The authors also noted that these ZnO NP treatments increased the percentage of carvone and decreased the percentage of limonene. In sweet marjoram, 100 mg L^-1^ ZnO NPs were the most effective application for improving EO % and EOY compared to the control, 50 mg L^-1^ ZnO NPs, and 100 mg L^-1^ of either nano-Fe or nano-Mn (El-Khateeb et al., 2020). Furthermore, spraying *Silybum marianum* plants with 1 g L^-1^ ZnO NPs notably boosted seed yield per feddan and total silymarin content in fruits (EL-Agouze et al., 2022). According to Al-Khazraji et al. (2025), the highest EO content in rosemary was observed with 20 mg L^-1^ ZnO NPs.

The results of the current study suggest that applying ZnO NPs improved the measured caraway traits across all NaCl levels compared with treatments without ZnO NPs. Zinc supplementation minimized the toxic effects of salinity by promoting chlorophyll synthesis and growth and by enhancing the antioxidant defense system (Al-Zahrani et al., 2021). Additionally, zinc protects membranes from oxidative damage by maintaining membrane integrity and permeability (Aravind and Prasad, 2004). Spraying ZnO NPs reduces membrane-bound NADPH oxidase activity and enhances SOD, CAT, and POD activities, thereby conferring salt resistance (Aktaş et al., 2006). The current study demonstrated that caraway exhibited good tolerance up to high NaCl level when sprayed with ZnO NPs. This finding aligns with Hassan (2019), who reported that caraway could tolerate salinity at 1000 and 2500 ppm after spraying with 400 mg L^-1^ humic acid. Zinc also reduces ROS production and protects membrane integrity, thereby decreasing the loss of essential osmolytes (Dehnavi et al., 2020).based on literatures ZnO NPs spraying improves plant growth and yield under salinity conditions by inducing cell signaling, thereby boosting gene expression, stress-responsive protein expression, antioxidant activity, and the accumulation of hormones and osmolytes. Zinc plays an essential role in the synthesis of phenolic and flavonoid compounds, which can aid in ROS scavenging under salinity conditions, thereby enhancing plant growth and development in amaranth (Sarker and Oba, 2018) and *Solanum lycopersicum* (Ali et al., 2022). Furthermore, zinc enhances nutrient and water absorption, promotes photopigment synthesis, protects leaf water content, and minimizes the absorption of damage ions (Na^+^ and Cl^-^) (Shao et al., 2023).

Nano-ZnO at 1 g L^-1^ increased the fresh and dry weights of *Dracocephalum maldavica* at 50 and 100 mM NaCl relative to untreated plants (Yaichi et al., 2025). These authors added that photosynthetic pigments and EO content were improved under such treatments with ZnO NPs and NaCl. Furthermore, ZnO NPs can aid salt-stressed plants in restoring cellular function in crop species (Weng et al., 2025). Spraying ZnO NPs during salinity stress improved growth efficacy by stimulating the electron transport chain and increasing antioxidant defense (Wang et al., 2019). Zn-mediated rises in growth under salinity stress is associated with membrane integrity, phospholipid build up, synthesis of protein, ROS removal, nutrient translocation, and restricted Na^+^ and Cl^-^ entry (Ali et al., 2022), which improves Mg uptake, an essential component of chlorophyll that enhances photosynthetic pigments (Amiri et al., 2016). Applying ZnO NPs decreases NADPH oxidative activity and photo-oxidation and increases CAT, POD, and SOD activities, thereby protecting membrane stability under salinity stress (Galal, 2019). Additionally, the timing of Zn application (plant age) is important for increasing plant salt tolerance; in this regard, Chattha et al (2023) mentioned that Zn application at the booting and milking stages was an important practice for enhancing rice growth, quality, and yield. Indeed, water deficiency impairs metabolic processes, thereby adversely affecting plant growth; however, Zn utilization promotes higher RWC and enhances nutrient uptake under salt stress by regulating water absorption and transport (Aravind and Prasad, 2004). Furthermore, the application of ZnO NPs reduced Cl^-^ and Na^+^ and increased N, P, K, and Zn, thereby enhancing plant development under salt stress (Amiri et al., 2016). Safari et al. (2021) revealed that the utilization of S and Zn increased P, K, and Zn content and reduced Na content of *Brassica napus* under salinity stress relative to the control. These results are matched with our results. The positive impacts of ZnO NPs on root length, STI, nutrient content, RWC, enzyme activity, and a decrease in proline content, Na, and Cl % under NaCl levels led to improved fruit yield, weight of 100 fruits, fruits TP and TC, EO %, and EOY/plant of the caraway plant, while the various combinations of the used NaCl and ZnO NPs concentrations had various impacts on EO compounds. The yield and composition of EO can vary with climate and habitat conditions (temperature, relative humidity, photoperiod, soil fertility), planting methods and harvesting stages, genetics, plant age, environmental stresses, and pest infestations (Barragan-Ferrer et al., 2016). These conditions directly affect the biosynthesis of secondary metabolites, or changes in other biological functions may have an indirect impact. Our results were supported by the study of Hendawy and Khalid (2005), which showed that the interaction of soil salinity and Zn utilization caused an increased reduction in proline level and boosted EOY of sage plants. They added that soil salinity at 2500 ppm with Zn increased v-thujone and 1,8-cineole, whereas the same treatment reduced thujone and camphor in the EO of sage compared with the control. Bibliographic evidence consistently interprets the reduction in proline following ZnO nanoparticle (ZnO NP) application as a biomarker of alleviated abiotic stress rather than suppressed defense. In maize (*Zea mays*) exposed to salinity, leaf proline rose from 0.55–0.59 to 0.61–0.65 µmol g^-^¹ FW, confirming osmotic stress activation. Foliar ZnO NPs markedly reduced proline to 0.15–0.17 µmol g^-^¹ FW. The authors explicitly stated that this reduction signals “alleviation of salt-induced osmotic and oxidative stress” and that the nanoparticles “directly mitigate stress levels, thereby decreasing the plant’s necessity to produce excess proline (Khan et al., 2026; Mahawar et al., 2025) documented that in radish (*Raphanus sativus*) under 150–300 mM NaCl, salinity elevated proline, anthocyanins, flavonoids, and antioxidant enzyme activities. ZnO NPs reversed these effects, lowering proline alongside other stress metabolites while restoring photosynthetic electron transport and PSII quantum yield—changes attributed to reduced oxidative stress rather than inhibited defense metabolism. The same review noted that, in maize under PEG-induced drought, chitosan-loaded ZnO NPs decreased proline by 5.5% and concomitantly reduced malondialdehyde (MDA) by 21.1%, confirming that proline decline tracked with reduced lipid peroxidation (Inam et al., 2024). In rice under severe salt stress, ZnO NPs similarly mitigated “salinity-induced proline over-accumulation” while lowering H_2_O_2_ and MDA (Mishra et al., 2025). In coriander (*Coriandrum sativum*) subjected to drought, plants mobilized non-enzymatic antioxidants, increasing DPPH scavenging, total phenolics, and flavonoids. Application of ZnO NPs significantly reduced these elevated antioxidant responses, indicating that the nanoparticles attenuated the underlying oxidative stress and thereby lessened the plant’s compensatory defense mobilization (Hanif et al., 2024). In *Echinacea purpurea* under lead toxicity, proline accumulation rose alongside MDA, H_2_O_2_, and antioxidant overactivation. Combined ZnO NP treatment alleviated Pb stress by decreasing proline, MDA, H_2_O_2_, and antioxidant overactivation simultaneously—again showing that proline normalization accompanied overall stress relief (Khan et al., 2026).The consistent pattern in recent literature is that ZnO NPs improve water retention, nutrient uptake, and photosynthetic efficiency, thereby reducing ROS generation at the source. Because the plant experiences less cellular damage, it downregulates compensatory proline synthesis. When proline reduction occurs alongside decreased MDA, H_2_O_2_, and ROS—and coincides with improved growth, relative water content, and chlorophyll retention—it should be interpreted as evidence of successful stress mitigation rather than defense inhibition.

The combinations of NaCl (0, 50, and 100 mM) and ZnO NPs (0 and 1000 mg L^-1^) led to improved EO biosynthesis and accumulation compounds of *Dracocephalum moldavica* (Yaichi et al., 2025). Wu and Ma (2025) on *Glycyrrhiza uralensis* reported that ZnO NPs not only improve salt tolerance but also redirect metabolic resources toward the biosynthesis of medicinal compounds. In conclusion, caraway can be treated with ZnO NPs to mitigate the adverse effects of salinity on growth, yield, EO productivity, and related attributes.

## 5 Conclusion

Salinity is one of the abiotic stresses that pose substantial impediments to sustainable agriculture. Nano-nutrients can ameliorate the detrimental effects of salinity on plants by modulating diverse physiological, chemical, and biochemical functions within plant cells. Caraway plants demonstrated complete survival across all tested NaCl concentrations, indicating notable tolerance and growth under low to medium NaCl concentrations; however, high NaCl levels had a significant negative impact relative to the other NaCl levels. The use of ZnO NPs, particularly at a concentration of 0.4 gL^-^¹, enhanced leaf relative water content (RWC), root length, chemical nutrient content, pigment concentrations, and enzyme activity in caraway plants subjected to the tested NaCl concentrations. Consequently, fruit yield, TP, and TC, essential oil (EO) productivity, and stress tolerance index (STI) were positively affected. Further study is required to elucidate the specific role of zinc (Zn) and its effects on nutrient signaling, hormone and osmolyte accumulation, nutrient uptake, and enzyme activity under salt stress. In conclusion, foliar spraying of ZnO NPs at 0.4 g L^-^¹ is a viable strategy for mitigating the toxic effects of salinity on caraway plants.

## Data Availability

The original contributions presented in the study are included in the article/supplementary material. Further inquiries can be directed to the corresponding author.
